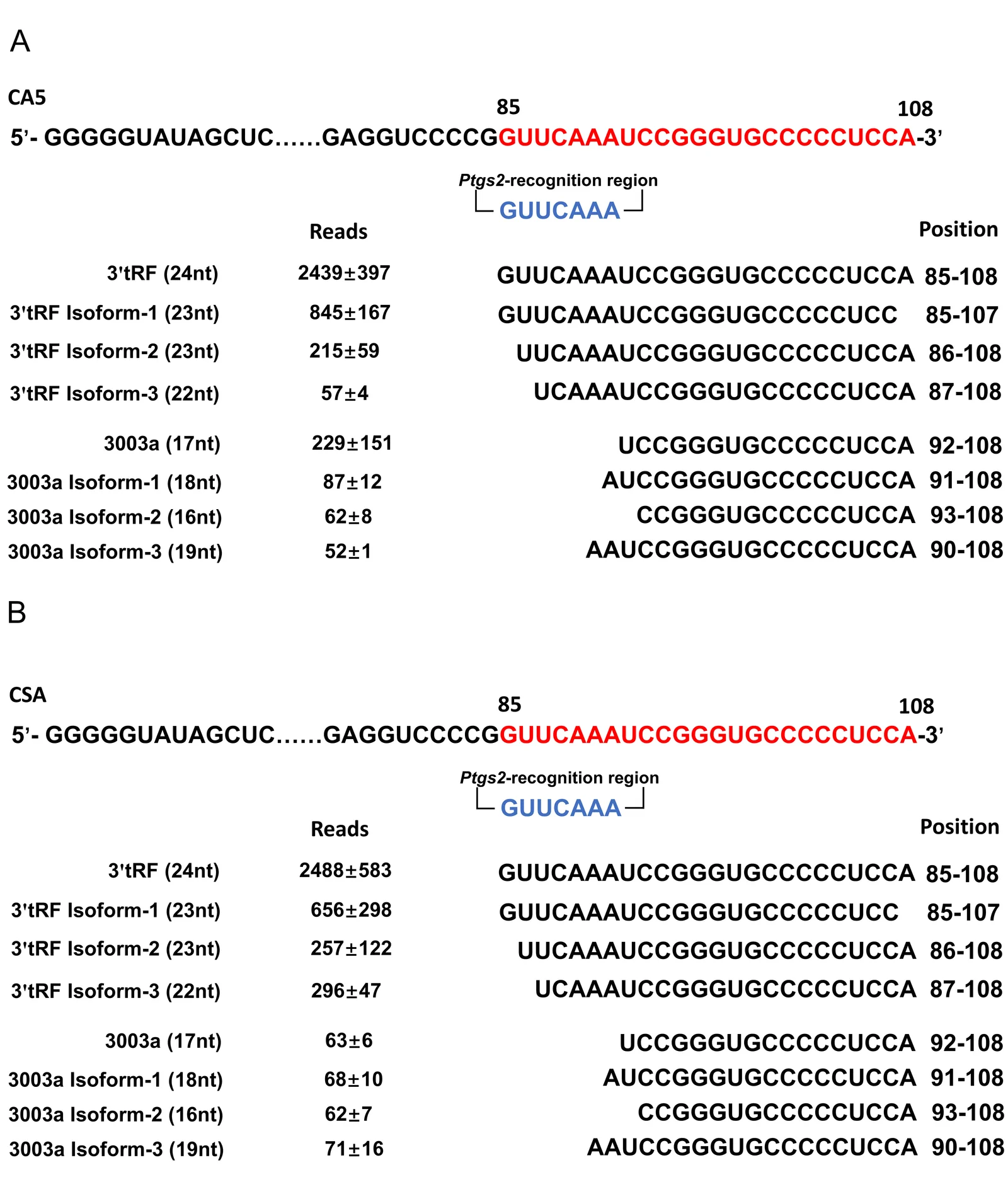# A tRNA^Cys^-aptamer chimera co-targeting ADAMTS5 and *Ptgs2* interrupts the ECM-pyroptosis feedback loop in osteoarthritis

**DOI:** 10.1016/j.ncrna.2026.10.003

**Published:** 2026-11-03

**Authors:** Bo Sang, Ye Tian, Kang Ru, Xuehao Wang, Suryaji Patil, Wenjin Zhong, Xinyao Jia, Zhihao Chen, Hui Li, Airong Qian

**Affiliations:** a Lab for Bone Metabolism, Xi'an Key Laboratory of Special Medicine and Health Engineering, Key Lab for Space Biosciences and Biotechnology, Research Center for Special Medicine and Health Systems Engineering, School of Life Science and Technology, Northwestern Polytechnical University, Xi'an, Shaanxi, 710072, China; b Department of Adult Joint Reconstruction, Honghui Hospital, Xi'an Jiaotong University, Xi'an, Shaanxi, 710054, China

**Keywords:** Osteoarthritis, ECM, tRNA chimera, ADAMTS5 aptamer, tRNA-derived fragment, *Ptgs2* (COX-2), NF-κB

## Abstract

Osteoarthritis (OA) is driven by a self-amplifying cycle of extracellular matrix (ECM) destruction and inflammatory cell death, yet disease-modifying therapies remain unavailable. Here, we engineer a multifunctional RNA chimera (CA5) by fusing a humanized tRNA^Cys^ anticodon stem-loop scaffold with an ADAMTS5 aptamer, thereby integrating protease inhibition with small RNA-like post-transcriptional regulation within a single RNA molecule. In IL-1β-stimulated chondrocytes, CA5 mitigates aggrecan and collagen degradation, suppresses inflammatory mediators, and robustly inhibits pyroptosis, exhibiting pronounced synergy between the tRNA^Cys^ scaffold and the aptamer domain. Consistent with these in vitro findings, intra-articular administration of CA5 in the destabilization of the medial meniscus (DMM) mouse model preserves articular cartilage, alleviates synovitis, and limits subchondral bone deterioration without detectable systemic toxicity. Mechanistically, structural and biophysical analyses demonstrate that CA5 adopts a stable G-quadruplex-containing architecture and binds ADAMTS5, enabling potent blockade of aggrecanase. Moreover, CA5 undergoes intracellular processing to release a conserved 3ꞌtRNA-derived fragment (3ꞌtRF) that directly targets the 3ꞌUTR of *Ptgs2* mRNA, leading to *Ptgs2* repression and subsequent attenuation of NF-κB signaling, thereby breaking the ECM-pyroptosis feedback loop. Collectively, CA5 represents a dual-action, single-molecule RNA therapeutic that couples aptamer-mediated ADAMTS5 inhibition with 3ꞌtRF-driven *Ptgs2* silencing to counteract OA pathogenesis, offering a modular strategy for next-generation OA interventions.

## 1 Introduction

Osteoarthritis (OA) is a degenerative joint disease characterized by progressive breakdown of the articular cartilage extracellular matrix (ECM) and chronic, low-grade inflammation, ultimately leading to persistent pain and irreversible disability [1], [2]. The continued absence of approved disease-modifying OA drugs (DMOADs) underscores that OA progression is driven by self-reinforcing catabolic and inflammatory programs that are difficult to reverse through single-node intervention [3], [4]. Therefore, elucidating the pathogenic mechanisms underlying OA and developing effective and safer therapeutic strategies remain urgent priorities.

Accumulating evidence suggests that OA progression is sustained by a reciprocal interplay between ECM breakdown and inflammatory signaling. Pro-inflammatory stimuli such as interleukin-1β (IL-1β) suppress ECM anabolism while inducing matrix-degrading enzymes, thereby progressively compromising cartilage structural integrity [5], [6], [7], [8]. In parallel, pyroptosis, a lytic inflammasome-dependent form of programmed cell death, has emerged as an important amplifier of joint inflammation in OA [9], [10]. Danger-associated and cytokine-associated signals activate the NOD-like receptor family pyrin domain-containing 3 (NLRP3) inflammasome in chondrocytes, resulting in caspase-1 activation, gasdermin D (GSDMD) cleavage, and release of pro-inflammatory cytokines such as IL-1β and IL-18 [11], [12]. These mediators further intensify synovitis and upregulate catabolic programs that accelerate ECM degradation [13]. Conversely, ECM disruption generates matrix-derived damage-associated molecular patterns (DAMPs) that sensitize chondrocytes to inflammasome activation and pyroptosis [14], [15], [16]. Together, these events form an “ECM-pyroptosis feedback loop” that drives progressive cartilage destruction and OA development, providing a strong rationale for therapeutic strategies designed to interrupt this pathogenic circuit [17], [18], [19], [20].

Aggrecan is a major structural component of cartilage that confers its gel-like properties and mechanical resilience [21], [22]. In OA, A Disintegrin and Metalloproteinase with Thrombospondin Motifs 5 (ADAMTS5) is a principal aggrecanase responsible for proteoglycan degradation, and its dysregulation disrupts ECM homeostasis and promotes cartilage damage, thereby establishing ADAMTS5 as an important therapeutic target [23], [24]. Importantly, ECM breakdown not only weakens cartilage biomechanics but also generates matrix-derived inflammatory cues that further aggravate the chondrocyte microenvironment. As a major mediator of aggrecan degradation, ADAMTS5 may contribute to the generation of matrix-derived DAMP-like signals during OA progression. These matrix-derived signals can engage innate immune pathways and may contribute to NLRP3 inflammasome priming, thereby facilitating pyroptotic responses [25], [26]. Thus, inhibition of ADAMTS5-driven aggrecanolysis may attenuate the ECM-pyroptosis feedback loop by reducing the burden of catabolic matrix fragments and the downstream amplification of inflammatory signaling, thereby limiting pyroptotic cell death. Consistent with this rationale, several DNA and RNA aptamers have been developed to target ADAMTS5. For example, G-quadruplex-forming DNA aptamers bind and inhibit ADAMTS5 with nanomolar affinity, and ADAMTS5-targeting aptamers have shown efficacy in preclinical models [27], [28].

The OA joint microenvironment is also characterized by sustained elevation of pro-inflammatory cytokines such as IL-1β and TNF-α, which drive chondrocytes and synovial cells toward an inflammatory and catabolic state through activation of the canonical NF-κB pathway, including IκBα phosphorylation and p65 nuclear translocation [29]. This signaling cascade amplifies inflammatory mediator expression and accelerates OA progression, while concurrently inducing Prostaglandin-Endoperoxide Synthase 2/Cyclooxygenase-2(PTGS2/COX-2), a central effector of inflammatory amplification in the osteoarthritic joint [30]. In parallel, transfer RNAs (tRNAs) can give rise to tRNA-derived small RNAs (tsRNAs) through regulated cleavage under diverse cellular conditions. Based on their cleavage patterns, these tsRNAs include 5ꞌtRFs, 3ꞌtRFs, tRNA halves (tiRNAs), and internal tRFs (i-tRFs) [31]. Increasing evidence indicates that tsRNAs participate in RNA modification, post-transcriptional gene regulation, protein synthesis, and other cellular processes, and are increasingly implicated in human disease, including OA [32]. Notably, tRF-3003a is enriched in healthy cartilage but progressively decreases in OA cartilage and correlates inversely with IL-6 levels, whereas restoration of tRF-3003a through tRNA^Cys^ overexpression or a tRF-3003a mimic suppresses IL-6 expression in chondrocytes, supporting a functional role for tRNA^Cys^-derived fragments in maintaining cartilage homeostasis and restraining OA-associated inflammation [33]. Additional studies have shown that tRF-5009A targets *mTOR* to regulate autophagy and cartilage degeneration, whereas tRF365 targets *IKBKB* to dampen NF-κB signaling and mitigate inflammatory programs in OA [34], [35]. More recently, tsRNA-12391 was reported to be downregulated in OA cartilage and to alleviate cartilage degeneration by promoting PINK1-associated mitophagy, further supporting the emerging role of tRNA-derived small RNAs in maintaining chondrocyte and cartilage homeostasis [36]. These observations support a broader role for tRNA-derived fragments as endogenous regulators of inflammatory signaling, cellular homeostasis, and cartilage degeneration in OA.

On the basis of these observations, we reasoned that a multifunctional RNA therapeutic capable of combining extracellular ADAMTS5 blockade with intracellular tRF-mediated post-transcriptional regulation might more effectively suppress both matrix catabolism and inflammatory amplification in OA. To test this concept, we developed a dual-function tRNA chimera, CA5, by fusing a humanized tRNA^Cys^ scaffold with an ADAMTS5-binding aptamer, thereby integrating extracellular aggrecanase inhibition with intracellular gene regulation within a single RNA molecule. Following cellular uptake, CA5 was processed to release a 3ꞌtRNA-derived fragment that repressed *Ptgs2* and attenuated NF-κB signaling, thereby jointly constraining matrix catabolism and inflammatory amplification. We evaluated CA5 in IL-1β-stimulated chondrocytes and in the DMM mouse model, and observed concomitant suppression of aggrecan loss, inflammatory outputs, and pyroptotic activation in vitro, together with preservation of cartilage and joint structure in vivo. Collectively, these findings provide proof of concept for a single RNA molecule with multiple therapeutic mechanisms that can interrupt the ECM-pyroptosis feedback loop sustaining OA progression, and support a modular framework for the development of next-generation disease-modifying interventions for complex degenerative diseases.

## 2 Materials and methods

### 2.1 Production of tRNA-aptamer chimera

The tRNA-ADAMTS5 aptamer chimeras (CA5 and MA5) and negative controls (CSA and MSA) were produced via an *E. coli* expression platform and FPLC purification method as described previously [37]. Endotoxin characterization of recombinant RNA products generated using this production platform has been reported previously [38]. In brief, synthetic expression plasmids (Tsingke, Beijing, China) encoding a humanized hybrid tRNA scaffold were overexpressed in HST08 cells. Total bacterial RNA was extracted and purified by fast protein liquid chromatography (FPLC), and the electrophoretic integrity and homogeneity of the purified RNA preparations were assessed by 8% denaturing urea-PAGE (Fig. S1B–E). Purified tRNA-aptamer chimeras were provided by RNALink Biological Technology Co., Ltd.

### 2.2 Isolation and culture of cells

Cartilage from C57BL/6 mouse (8∼10 days old) was dissected under sterile conditions, rinsed with PBS containing 1% penicillin/streptomycin, minced into small fragments, and digested with 0.2% collagenase II at 37°C for 6 h. The released cells were passed through a 70 μm cell strainer, washed, and cultured in DMEM/F12 medium (Gibco, USA) supplemented with 10% FBS and 1% penicillin-streptomycin at 37°C in 5% CO_2_. Medium was replaced every 2 days, and cells at passages 1∼3 were used for subsequent experiments. ATDC5 cells were maintained in DMEM medium (Gibco, USA) containing 10% FBS and 1% penicillin-streptomycin in a humidified incubator at 37°C with 5% CO_2_.

### 2.3 Cell viability assay

Primary chondrocytes (96-well plates) were cultured to 80∼90% confluence and exposed to tRNA-aptamer chimeras (0∼100 nM) delivered by polyvinylamine (PVAm) in DMEM/F12 medium with 3% FBS for 6 h. After replacing with complete medium, cells were incubated for 48 h. Viability was quantified using CCK-8 (10 μL/well; Biosharp, Hefei, China) following 4 h incubation at 37°C, and absorbance was read at 450 nm.

### 2.4 Reverse transcription quantitative real-time PCR (RT-qPCR)

Primary mouse chondrocytes were plated in 12-well plates. To establish OA-like stimulation, cells were exposed to IL-1β (10 ng/mL) for 24 h, followed by transfection with tRNA-aptamer chimeras (25 nM) as described above. Total RNA was isolated using the E.Z.N.A. Total RNA Kit II (Omega Bio-tek, Norcross, GA, USA). First-strand cDNA was generated with HiScript II Q RT SuperMix (Vazyme, China) using random hexamer primers. RT-qPCR was performed with SYBR Green Master Mix (Vazyme, China) on a CFX96 Touch Real-Time PCR Detection System (Bio-Rad, Hercules, CA, USA) using gene-specific primers. GAPDH served as the reference gene, and relative mRNA abundance was calculated by the 2^−ΔΔCt^ method. The primer sequences are listed in Table S1.

### 2.5 Toluidine blue and Safranin-O staining

Primary murine chondrocytes were plated in 48-well plates and subjected to pretreatment/transfection as described above. Cells were washed twice with PBS and fixed with 4% paraformaldehyde for 20 min at room temperature. Fixed cells were then incubated with either Toluidine Blue or Safranin-O solution (Solarbio, Beijing, China) for 40 min in the dark, followed by rinsing with ultra-pure water. Stained plates were scanned using a flatbed scanner.

### 2.6 Western blot

Primary murine chondrocytes were seeded in 6-well plates and subjected to pretreatment and transfection as described above. Cells were rinsed with ice-cold PBS and lysed on ice in RIPA buffer (Beyotime, Shanghai, China) supplemented with protease inhibitors. Protein concentrations were determined using a BCA assay kit (Beyotime, Shanghai, China). Equal amounts of protein (30 μg per lane) were separated on 10% SDS-PAGE gels and electro-transferred onto 0.22 μm PVDF membranes. Membranes were blocked in 5% non-fat milk prepared in TBST for 1 h at room temperature, then incubated overnight at 4°C with primary antibodies against COL2A1 (1:1000; 28459‐1‐AP; Proteintech, Wuhan, China), IL-6 (1:1000; 21865-1-AP; Proteintech, Wuhan, China), PTGS2 (1:1000; 12375-1-AP; Proteintech, Wuhan, China), NLRP3 (1:1000; ET1610-93; HUABIO, Hangzhou, China), ASC (1:3000; A24165; Abclonal Wuhan, China), GSDMD (1:1000; #AF4012; Affinity Biosciences, Changzhou, China), GAPDH (1:20000; 10494-1-AP; Proteintech, Wuhan, China), β-actin (1:50000; 66009-1-Ig; Proteintech, Wuhan, China), p65 (1:2000; 10745-1-AP; Proteintech, Wuhan, China), Phospho-p65 (Ser536) (1:2000; 80379-2-RR; Proteintech, Wuhan, China), IκBα (1:2000; 10268-1-AP; Proteintech, Wuhan, China), Phospho-IκBα (1:2000; 82349-1-RR; Proteintech, Wuhan, China). After TBST washing, membranes were incubated with HRP-conjugated secondary antibodies for 2 h at room temperature. Signals were developed using ECL substrate (Biosharp, Hefei, China) and captured on a Tanon 4600SF imaging system (Tanon Science, Shanghai, China).

### 2.7 ELISA assays for sGAG and IL-18

sGAG and IL-18 levels were quantified using ELISA kits (Jiangsu Jingmei Biological Technology Co., Yancheng, China) following the supplier's instructions, and absorbance was read at 450 nm on a microplate reader (Synergy HT, Bio-Tek, USA). To prepare samples, primary mouse chondrocytes were cultured in 6-well plates and processed as described above. For sGAG analysis, cells were incubated with papain extraction buffer (100 μg/mL papain in Na_2_HPO_4_/NaH_2_PO_4_ buffer, pH 6.4) at 65°C for 3 h, then clarified by centrifugation at 10,000 × *g* for 10 min at 2-8°C and the supernatant was collected. For IL-18, conditioned media were harvested and cleared by centrifugation (3000 × *g*, 10 min) prior to ELISA.

### 2.8 Chou-Talalay combination index (median-drug effect analysis)

Synergistic, additive, or antagonistic interactions between the tRNA^Cys^ scaffold and the ADAMTS5 aptamer were quantified using the Chou-Talalay median-effect framework. Combination Index (CI) values were calculated in CompuSyn software (ComboSyn, Inc.), where CI < 1, CI = 1, and CI > 1 indicated synergism, additivity, and antagonism, respectively [39]. To enable CI computation, dose–response measurements were transformed into an effectiveness metric and then converted to the fraction affected (Fa). Specifically, Effectiveness% was defined as: Effectiveness% = (C_treatment_ − C_IL-1β_)/(C_normal_ − C_IL-1β_) × 100%, where C_normal_ denotes the aggrecan concentration in untreated control cells, C_IL-1β_ represents the aggrecan concentration after IL-1β challenge (10 ng/mL, 24 h), and C_treatment_ corresponds to the concentration measured after treatment with the indicated constructs (CA5, CSA, MA5). Aggrecan, a major cartilage proteoglycan and a direct substrate of ADAMTS5, was selected as the functional readout for extracellular matrix (ECM) preservation and ADAMTS5 inhibition. Primary mouse chondrocytes were primed with IL-1β (10 ng/mL) for 24 h and then transfected with individual constructs (CA5, CSA, MA5) at escalating total concentrations (1.5, 3, 6, 12, 25, and 50 nM) for 48 h. Cells were washed with ice-cold PBS and lysed on ice in RIPA buffer (Beyotime, Shanghai, China) containing protease inhibitors. Lysates were collected by scraping and clarified by centrifugation (10,000×*g*, 10 min, 2–8°C). Supernatants were used to quantify aggrecan using a commercial ELISA kit (Jiangsu Jingmei Biological Technology Co., Yancheng, China) following the manufacturer's instructions, with absorbance measured at 450 nm.

### 2.9 Immunofluorescence

ATDC5 cells or primary mouse chondrocytes were plated in 24-well plates and subjected to the treatments outlined below. Cells were then subjected to a conventional immunofluorescence workflow. Briefly, samples were fixed in 4% paraformaldehyde for 15 min at room temperature, permeabilized with 0.1% Triton X-100 for 5 min, and blocked with 5% bovine serum albumin for 1 h (all at room temperature). Primary antibodies were applied overnight at 4°C, followed by incubation with Alexa Fluor 488 or Cy3 labeled secondary antibodies (1:200) for 2 h at room temperature. Nuclei were counterstained with DAPI (1 μg/mL). Images were acquired using either an inverted fluorescence microscope or a confocal laser scanning microscope (CLSM), as specified for each experiment.

To examine aptamer-binding specificity through competition with an anti-ADAMTS5 antibody, ATDC5 cells (all groups except the blank control) were first transfected with an ADAMTS5 overexpression plasmid for 48 h. After fixation and blocking, cells in the MSA, CA5, CSA, and MA5 groups were incubated with the corresponding tRNA-aptamer chimeras (100 nM) for 2 h. Thereafter, all samples were immunostained with anti-ADAMTS5 antibody (1:100; bs-3573R; Bioss, Beijing, China). A decrease in anti-ADAMTS5 fluorescence intensity was interpreted as evidence of successful ADAMTS5 aptamer binding via epitope competition. Imaging was performed on an inverted fluorescence microscope (Observer 3, Zeiss, Germany).

For inflammasome-related markers, primary mouse chondrocytes were treated using the same experimental grouping as described for Western blotting and RT-qPCR. Cells were stained with anti-NLRP3 (1:100; ET1610-93; HUABIO, Hangzhou, China) or anti-CASPASE1 (1:50; SC-398715; Santa Cruz, Dallas, USA), and fluorescence signals were visualized by CLSM (Leica SP8, Germany).

Primary chondrocytes were pretreated with IL-1β for 24 h and subsequently transfected with the indicated tRNA-aptamer chimeras or chemically synthesized 3ꞌtRF and ADAMTS5 aptamer (A5) (GenePharma, Shanghai, China) for an additional 48 h. Cells were then incubated with an anti-p65 primary antibody (1:100; 10745-1-AP; Proteintech, Wuhan, China). Fluorescence images were captured using CLSM (Leica TCS SP8, Germany) to evaluate NF-κB p65 nuclear translocation.

### 2.10 Transmission electron microscopy analysis (TEM)

Primary mouse chondrocytes were pretreated and transfected as described above. Cells were collected by centrifugation (1000× *g*, 10 min) to form compact pellets, which were immediately fixed in 2.5% glutaraldehyde for 4 h at 25°C. Pellets were embedded in 1% agarose, rinsed in 0.1 M phosphate buffer (pH 7.4), and post-fixed with 1% osmium tetroxide. After thorough buffer washes, samples were dehydrated through a graded ethanol series, infiltrated, and embedded in resin. Ultrathin sections (70∼90 nm) were cut and sequentially contrasted with 2% uranyl acetate and lead citrate, air-dried at 25°C, and examined using TEM (JEM1400, Tokyo, Japan).

### 2.11 Circular dichroism (CD) spectroscopy for G-quadruplex

Representative tRNA-ADAMTS5 aptamer chimeras were prepared in a NaCl/CaCl_2_ containing buffer (100 mM NaCl, 10 mM CaCl_2_). CD measurements were performed on a JASCO J-810 spectropolarimeter by scanning from 200 to 350 nm at a rate of 120 nm/min (0.5 s per point; 1 nm bandwidth) using a 10 mm pathlength cuvette. Spectra were subsequently smoothed and processed in Origin 9.0.

### 2.12 ITC analysis of aptamer affinity

Aptamer-ADAMTS5 interactions were quantified by isothermal titration calorimetry (ITC), which measures the heat released/absorbed upon complex formation without the need for labels [40]. Recombinant ADAMTS5 protein (9.0 μM; 200 μL) was loaded into the sample cell, and tRNA-aptamer chimeras (100 μM) prepared in the identical buffer were placed in the syringe. Titrations were performed at 25°C with continuous stirring at 300 rpm, consisting of 20 sequential injections (3 μL each) separated by 180 s. Integrated heat pulses were fitted using a “one set of sites” binding model to derive the dissociation constant (*Kd*).

### 2.13 OA mouse model and intra-articular treatment

Male C57BL/6 mice (8 weeks, 18–20 g; Beijing Vital River, China) were acclimated for 7 days and maintained at 25°C and 55–60% humidity with ad libitum food and water. OA was induced by DMM surgery in the right knee through transection of the medial meniscotibial ligament; sham animals underwent arthrotomy without transection. On day 30 post-DMM, mice were randomized into six groups (Sham, PBS, MSA, CA5, CSA, MA5; n = 6/group). tRNA-aptamer chimeras were formulated with CAP-PVAm-PLGA (polymer: RNA = 1:1, w/w) and injected intra-articularly (10 μL; 0.5 mg/kg) every 5 days for 30 days [41]. Serum collected at sacrifice was analyzed for tumor necrosis factor-α (TNF-α) and interleukin-6 (IL-6), together with serum biochemistry parameters including aspartate aminotransferase (AST), alanine aminotransferase (ALT), blood urea nitrogen (BUN), creatinine (CREA), and uric acid (UA). All procedures were approved by the Laboratory Animal Ethics (No: 202301106) and Welfare Committee of Northwestern Polytechnical University (Xi'an, China) and followed the Guide for the Care and Use of Laboratory Animals.

### 2.14 Histopathological staining

At the study endpoint, knee joints were harvested for histological analysis. Samples were fixed in 4% paraformaldehyde for 24 h, decalcified in 10% EDTA for 1 week, and embedded in paraffin. Serial sagittal sections (5 μm) were obtained and stained with hematoxylin and eosin (H&E) and Safranin O/Fast Green to evaluate cartilage morphology and proteoglycan depletion. To assess potential systemic toxicity of the tRNA-ADAMTS5 aptamer chimeras, liver and kidney tissues were also subjected to H&E staining. Cartilage matrix and inflammatory status were further examined by immunohistochemistry for COL2A1, AGGRECAN, and IL-6. Images were acquired using an upright light microscope, and OA severity was graded using the OARSI scoring system and Mankin score.

### 2.15 Micro-CT imaging

Fixed knee joints were analyzed by micro-CT system (VivaCT80; Scanco Medical AG, Switzerland) to characterize trabecular microarchitecture. Specimens were fixed in 4% paraformaldehyde, secured on the sample holder, and scanned at 10.4 μm isotropic resolution under the following settings: 70 kV, 114 μA, and 205 ms integration time. Image acquisition and morphometric quantification were performed using Scanco Evaluation Software, and three-dimensional renderings were generated with Ray Tracer. Structural indices, including bone volume fraction (BV/TV), trabecular number (Tb.N), trabecular separation (Tb.Sp), and trabecular thickness (Tb.Th), were calculated.

### 2.16 RNA-seq and computational analysis

Total RNA from pretreated/transfected primary mouse chondrocytes was extracted with TRIzol and quality-checked using an Agilent 2100 Bioanalyzer. Poly(A) + mRNA was enriched with oligo (dT) magnetic beads, fragmented, and converted to double-stranded cDNA using random hexamers. Sequencing libraries were generated by end repair, A-tailing, adaptor ligation, size selection, PCR enrichment, and purification. Libraries were quantified by Qubit and RT-qPCR, evaluated for insert size on the Bioanalyzer, pooled based on effective molarity, and sequenced on an Illumina platform.

### 2.17 Small RNA-seq and tRNA-aptamer chimera derived fragment mapping

Total RNA from pretreated/transfected primary mouse chondrocytes was extracted with TRIzol. Small RNA libraries were constructed by 3′/5′ adapter ligation, reverse transcription, and PCR enrichment, with 18∼40 nt inserts size selected, purified, QC-verified (insert size and molarity), pooled equimolarly, and sequenced on an Illumina platform. Adapter-trimmed and length-filtered reads were aligned using Bowtie to a reference containing full tRNA-aptamer chimera sequences, thereby defining chimera-derived fragments and their positional distributions.

### 2.18 Dual-luciferase reporter assay

To determine the regulatory relationship between the 3ꞌtRF and *Ptgs2* mRNA, a dual-luciferase reporter assay was performed in primary chondrocytes. The *Ptgs2* 3ꞌ-UTR containing either the predicted 3ꞌtRF binding site or its mutant variant was cloned into the luciferase reporter plasmid. Primary chondrocytes were co-transfected with the *Ptgs2* 3ꞌ-UTR reporter plasmid (wild-type or mutant), along with CA5, CSA, the corresponding negative control (MSA), and a 3ꞌtRF mimic or its respective negative control (NC). Forty-eight hours post-transfection, cells were harvested. Firefly and Renilla luciferase activities were measured using the Dual-Luciferase Reporter Gene Assay Kit (Beyotime, Shanghai, China) according to the manufacturer's protocol, with detection performed on a microplate reader (Synergy HT, BioTek, USA). Firefly luciferase activity was normalized to Renilla luciferase activity for each sample.

### 2.19 Statistical analysis

Data are presented as mean ± SD. Statistical analyses were performed in GraphPad Prism 8.0 and Origin. Two-group comparisons used unpaired two-tailed Student's *t*-tests; multiple groups used one-way ANOVA with appropriate post hoc tests. *P* < 0.05 was considered significant. Exact *n*, statistical tests, and *P* values are reported in figure legends.

## 3 Results

### 3.1 A tRNA^Cys^-ADAMTS5 aptamer chimera confers synergistic protection against IL-1β-induced cartilage matrix loss

We selected tRNA^Cys^, which is downregulated in osteoarthritic cartilage [33], as the scaffold and fused it to a validated ADAMTS5-targeting aptamer to generate the chimeric construct CA5 (Fig. 1A). To evaluate scaffold specific effects, we engineered a control chimera by fusing tRNA^Cys^ to an inert Sephadex binding aptamer, termed CSA. In parallel, two additional constructs based on a bacterial tRNA scaffold were included: MA5, in which the functional ADAMTS5 aptamer was fused to bacterial tRNA^Met^ (bt-tRNA^Met^) as an aptamer matched comparator, and MSA, in which bt-tRNA^Met^ was fused to the inert Sephadex aptamer as a full-length negative control. All four chimeric RNAs (CA5, CSA, MA5, and MSA) were highly expressed in *E. coli* and subsequently purified by FPLC (Fig. S1A–E). Following transfection into primary chondrocytes, robust intracellular accumulation was observed (Fig. S2A–B), and no detectable cytotoxicity was noted at concentrations up to 100 nM (Fig. S2C–F).

**Fig. 1:**
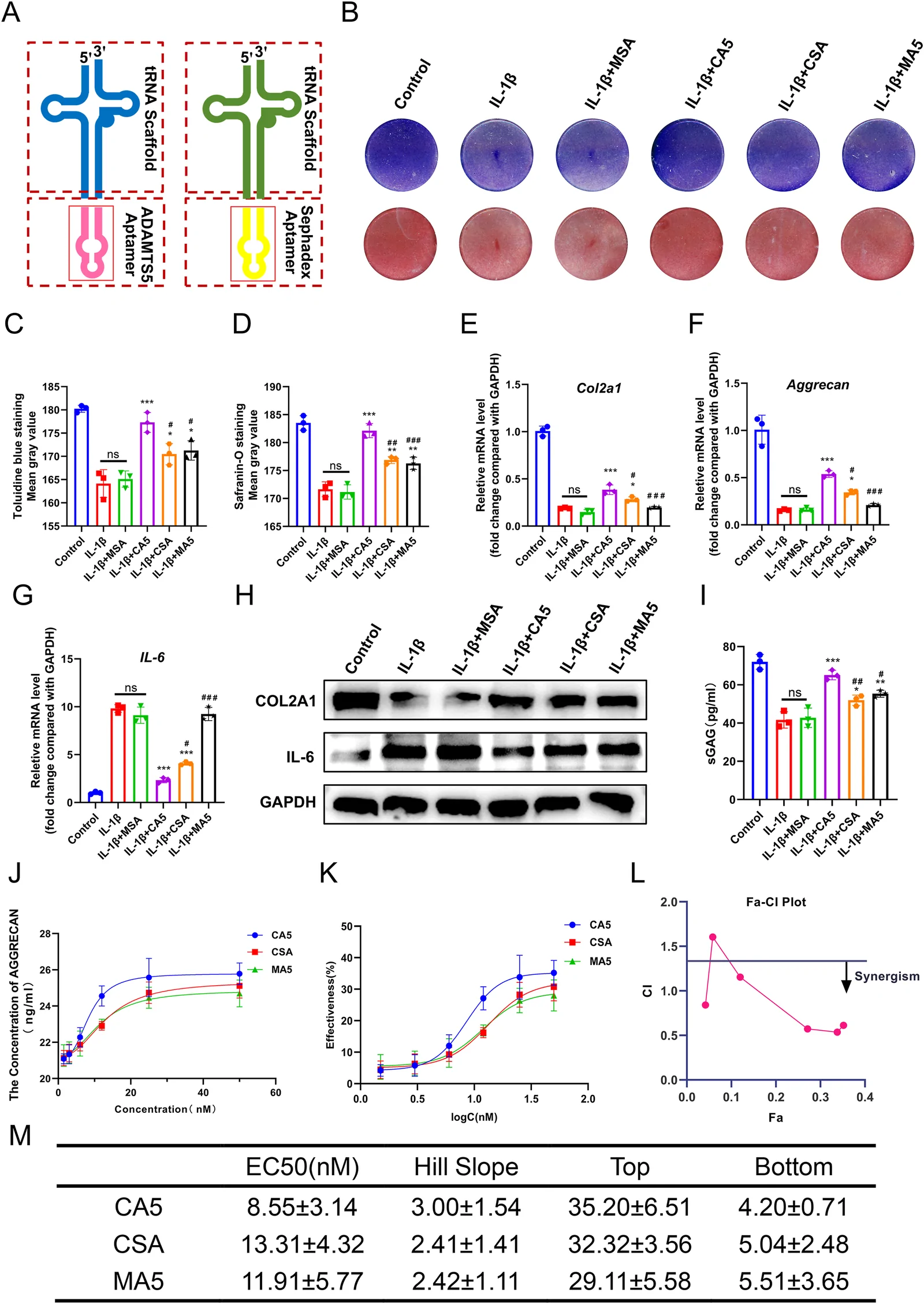
**Chondroprotection by tRNA^Cys^-aptamer chimera in IL-1β stimulated primary chondrocytes.** A) Scheme of tRNA-aptamer chimera constructed by fusing tRNA scaffold with either an ADAMTS5-targeting or a non-functional control aptamer. B-D) Toluidine blue and Safranin O staining in primary chondrocytes. E-G) RT-qPCR analysis of cartilage matrix genes (*Col2a1*, *Aggrecan*) and inflammatory genes (*Il-6*). H) WB analysis of COL2A1 and IL-6 protein levels. I) Total sGAG content quantified by ELISA. J) AGGRECAN levels were quantified by ELISA as a readout of ECM preservation and ADAMTS5 inhibition. K) Dose-response curves for the indicated tRNA-aptamer chimera. L) Fa-CI plot for combination analysis (Fa: fraction affected; CI: combination index). M) Pharmacodynamic parameters estimated by nonlinear regression of dose-response data. Data were presented as mean ± SD (n = 3). Statistical analysis was performed using one-way ANOVA followed by Tukey's test. *P < 0.05 **P < 0.01 and ***P < 0.001 vs. the IL-1β group. #*P* < 0.05 ##*P* < 0.01 and ###*P* < 0.001 vs. the CA5 group.

In IL-1β-stimulated primary chondrocytes, CA5, CSA, and MA5 all increased Toluidine Blue and Safranin-O staining intensity relative to the IL-1β and MSA controls, with CA5 producing the most pronounced restoration of matrix staining (Fig. 1B–D). Consistently, CA5 markedly rescued the mRNA expression of the cartilage anabolic markers *Col2a1* and *Aggrecan* while suppressing the inflammatory mediator *IL-6* (Fig. 1E–G); these effects were further corroborated at the protein level, showing recovery of COL2A1 and reduction of IL-6 (Fig. 1H). Quantification of sulfated glycosaminoglycan (sGAG) further confirmed CA5-mediated matrix preservation, whereas CSA and MA5 provided only modest protection compared with CA5 (Fig. 1I).

Dose-response analyses revealed that CA5 enhanced AGGRECAN production in a concentration-dependent manner and consistently outperformed CSA and MA5 across the 1.5-50 nM range (Fig. 1J). Accordingly, CA5 exhibited the lowest EC50 (8.55 ± 3.14 nM), compared with CSA (13.31 ± 4.32 nM) and MA5 (11.91 ± 5.77 nM), indicating superior potency (Fig. 1K), with additional pharmacodynamic parameters summarized (Fig. 1M). Chou-Talalay analysis further supported strong synergy between the tRNA^Cys^ scaffold and the ADAMTS5 aptamer module, yielding combination indices (CI) < 1 (Fig. 1L). Notably, despite lacking an ADAMTS5-binding domain, CSA still attenuated ECM loss, suggesting an aptamer-independent scaffold effect. Moreover, the weaker activity of MA5 relative to CA5 highlights the functional advantage conferred by the tRNA^Cys^ scaffold in cooperation with the ADAMTS5 aptamer, collectively resulting in more robust ECM protection.

### 3.2 tRNA-aptamer chimera suppresses IL-1β-induced inflammasome activation and chondrocyte pyroptosis

Given that inflammasome-driven pyroptosis amplifies cartilage catabolism through IL-1β/IL-18 release, we next examined whether the tRNA-aptamer chimeras could dampen IL-1β evoked pyroptotic cascades in primary chondrocytes. RT-qPCR profiling showed that IL-1β robustly induced *Nlrp3*, *Caspase1*, and *Asc* transcripts, whereas CA5 and CSA significantly curtailed these increases; MA5 produced a partial reduction that did not reach statistical significance (Fig. 2A–C). At the protein level, WB analysis confirmed elevated NLRP3 and ASC together with increased GSDMD-N after IL-1β challenge, while total GSDMD remained largely stable; each tRNA-aptamer chimera attenuated these pyroptosis-associated changes (Fig. 2D). Consistent with these findings, immunofluorescence revealed strong induction of NLRP3 and CASPASE1 in IL-1β-treated cells, which was markedly reduced upon treatment with CA5, CSA, or MA5 (Fig. 2E and F).

**Fig. 2:**
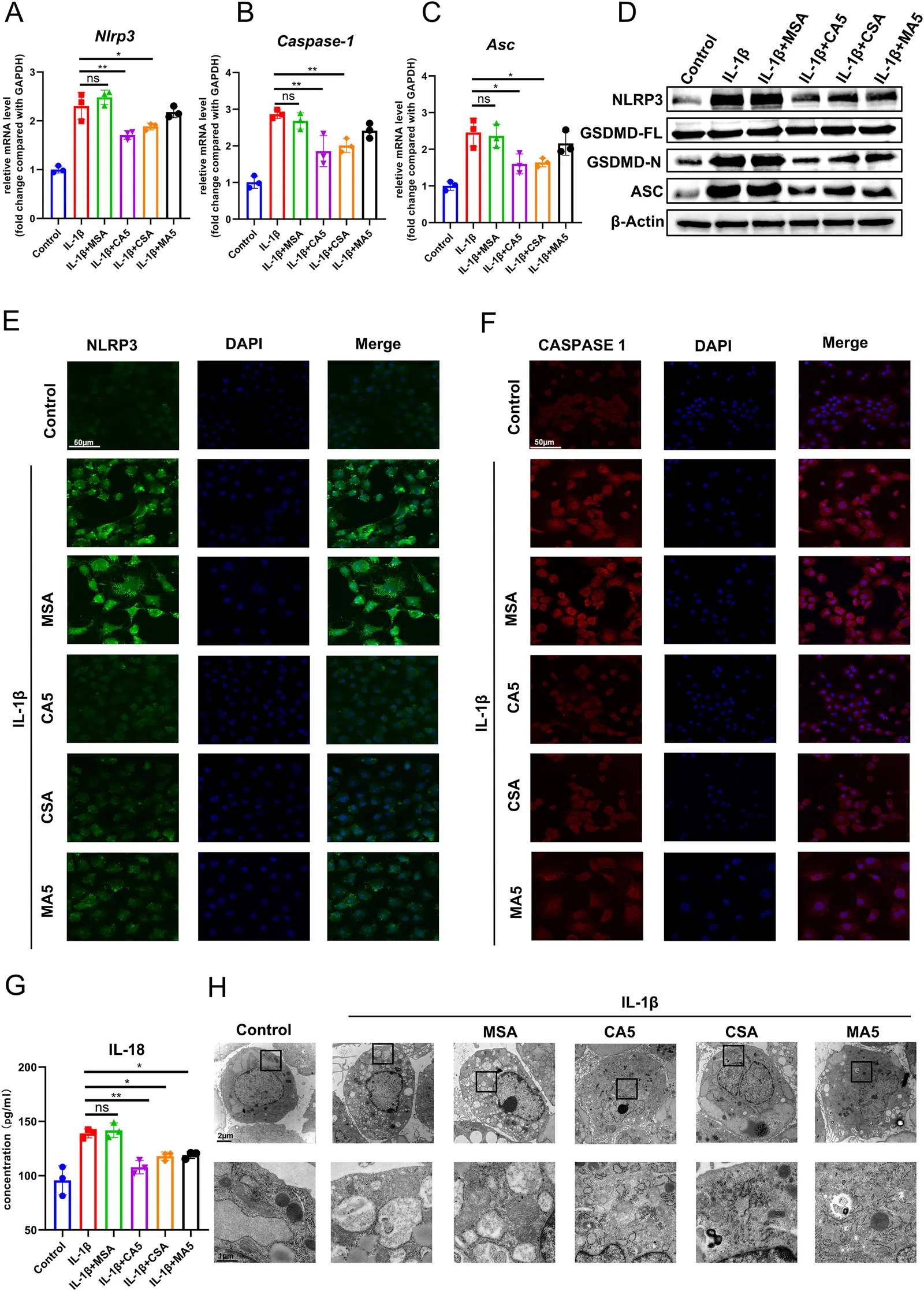
**tRNA-aptamer chimeras attenuate IL-1β induced pyroptosis in primary chondrocytes.** A-C) RT-qPCR analysis of inflammasome-related transcripts (*Nlrp3*, *Caspase1*, *Asc*) in IL-1β stimulated primary chondrocytes following transfection with the indicated tRNA-aptamer chimeras. D) WB analysis of NLRP3, ASC, full-length GSDMD, and GSDMD-N. E-F) Confocal immunofluorescence images showing NLRP3 and CASPASE1 staining in primary chondrocytes under the indicated conditions. G) Secreted IL-18 in culture supernatants quantified by ELISA. H) Representative TEM images illustrating ultrastructural changes in primary chondrocytes after IL-1β challenge with or without treatment of tRNA-aptamer chimeras. Data were presented as mean ± SD (n = 3). Statistical analysis was performed using one-way ANOVA followed by Tukey's test. *P < 0.05 **P < 0.01 and ***P < 0.001 vs. the IL-1β group.

Functionally, IL-1β substantially increased extracellular IL-18, a hallmark output of GSDMD pore formation, and this elevation was suppressed by all three constructs as measured by ELISA (Fig. 2G). TEM analysis further corroborated these biochemical readouts. IL-1β exposure elicited characteristic pyroptotic morphology, including cellular swelling, membrane ballooning, extensive vacuolization, and mitochondrial edema, whereas tRNA-aptamer chimera-treated chondrocytes largely preserved membrane integrity and organelle architecture (Fig. 2H). Notably, MA5 still alleviated pyroptotic phenotypes, implying that extracellular ADAMTS5 blockade and consequent ECM stabilization can indirectly restrain inflammasome activation. Conversely, ECM protection observed with scaffold-driven constructs in the absence of direct ADAMTS5 binding supports reciprocal crosstalk between matrix loss and pyroptosis. Together, these results are consistent with an ECM-pyroptosis feedback loop, which is most effectively interrupted by CA5 integrating both scaffold and aptamer-derived functions.

### 3.3 Intra-articular delivery of tRNA-aptamer chimeras attenuates DMM-induced osteoarthritis and preserves cartilage and subchondral bone architecture

To facilitate intra-articular delivery of the chimeric tRNAs in vivo, we formulated the constructs using our previously established cartilage-targeting carrier, CAP-PVAm-PLGA [41]. In the DMM model, joints receiving tRNA-aptamer chimeras exhibited markedly reduced osteoarthritic deterioration compared with PBS or the full-length negative control (MSA) (Fig. 3A). Histological assessment of the medial femoral condyle and tibial plateau showed evident surface chondrocyte depletion in PBS-and MSA-treated mice, whereas this damage was substantially mitigated following tRNA-aptamer chimera administration (Fig. 3B). Consistent with improved matrix retention, Safranin O staining intensity and cartilage thickness were restored, with CA5 producing more pronounced protection than CSA or MA5 (Fig. 3C). Accordingly, cartilage degeneration grades, quantified by OARSI and Mankin scoring, were significantly reduced in treated joints, and CA5 conferred the greatest improvement (Fig. 3H and I). Given the pivotal role of subchondral bone in supporting cartilage, we next analyzed tibial microarchitecture by micro-CT. PBS and MSA treated joints displayed severe plateau abrasion, osteophyte development, and disrupted trabecular networks (Fig. 3D). In contrast, chimeric tRNA treatment limited osteophyte formation and preserved trabecular organization, accompanied by significant improvements in Tb.N, Tb.Th, Tb.Sp, and BV/TV, again with the strongest effects observed for CA5 (Fig. 3J–M). Immunohistochemistry further supported the joint-protective effects of the tRNA-aptamer chimeras. COL2A1 and AGGRECAN staining was markedly reduced in the PBS and MSA groups but was substantially restored following treatment with CA5, CSA, and MA5 (Fig. 3E and F). In parallel, IL-6 immunoreactivity was markedly elevated in PBS and MSA treated joints and was attenuated after administration of the chimeric constructs (Fig. 3G). Collectively, these changes are consistent with reduced matrix breakdown, enhanced ECM retention, and attenuated local inflammatory signaling. Systematic safety analyses indicated no detectable treatment-related toxicity or inflammatory activation. Liver and kidney histology were normal (Fig. S3A–B), serum biochemistry parameters (ALT, AST, BUN, CREA, UA) stayed within physiological ranges (Fig. S4A–E), and circulating IL-6 and TNF-α levels were unchanged (Fig. S4F–G).

**Fig. 3:**
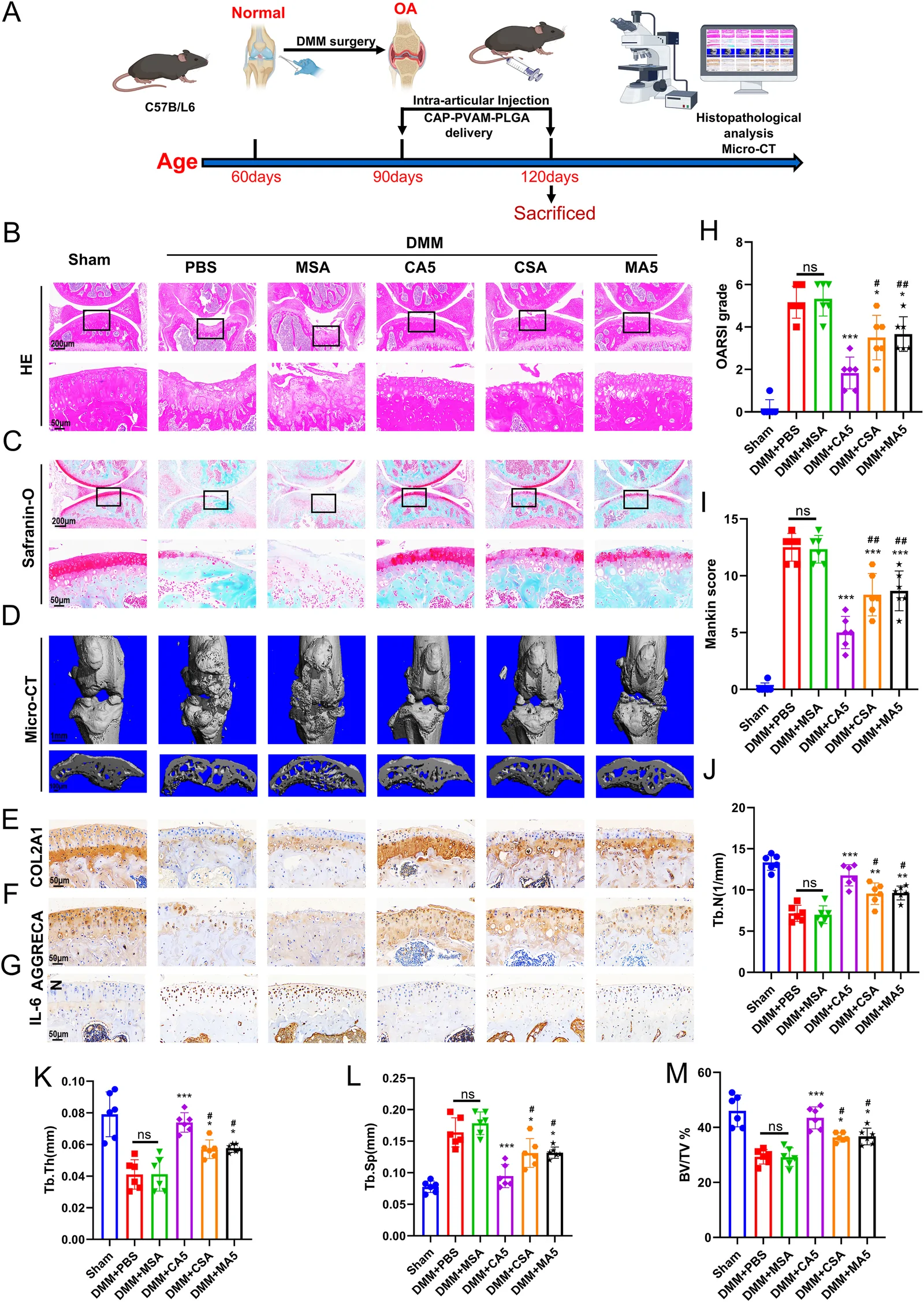
**Histological and micro-CT evidence of joint protection by intra-articular tRNA-aptamer chimeras in the DMM model.** A) Schematic overview of the DMM surgery and intra-articular treatment schedule. B) Representative H&E-stained sections of knee joints after DMM surgery to assess cartilage morphology and tissue integrity. The upper panels highlight cartilage damage in the weight-bearing region of the medial tibial plateau, whereas the lower panels show the contact region between the medial tibial plateau and medial femoral condyle and illustrate chondrocyte morphology. C) Representative frontal sections stained with Safranin-O/Fast Green from sham or DMM operated mice, indicating proteoglycan loss and cartilage erosion. D) Representative 3D reconstructions of the articular surface (upper) and subchondral bone (lower) obtained by micro-CT. Regions of interest (ROIs) were delineated across serial frontal image stacks and rendered for three-dimensional visualization. E-G) Immunohistochemical staining of COL2A1, AGGRECAN and IL-6 in articular cartilage following treatment with tRNA-ADAMTS5 chimeras. H-I) Quantification of OA severity by OARSI and Mankin scoring. J-M) Micro-CT based quantification of tibial subchondral trabecular parameters after 30 days of treatment, including trabecular number (Tb.N), trabecular thickness (Tb.Th), trabecular separation (Tb.Sp), and bone volume fraction (BV/TV). Data were presented as mean ± SD (n = 6). Statistical analysis was performed using one-way ANOVA followed by Tukey's test. *P < 0.05 **P < 0.01 and ***P < 0.001 vs. the PBS group. *#P* < 0.05 and *##P* < 0.01 vs. the CA5 group.

Together, these findings demonstrate that intra-articular delivery of chimeric tRNAs, particularly CA5, preserves both articular cartilage and subchondral bone structure in DMM-induced OA without evident systemic adverse effects, supporting a disease-modifying potential in vivo.

### 3.4 CA5 and MA5 adopt G-quadruplex structures and bind ADAMTS5 with sub-micromolar affinity

G-quadruplex (G4) motifs are known to stabilize RNA structures and enhance resistance to nuclease degradation, and have been reported as a defining structural feature of the ADAMTS5 aptamer required for target recognition [27], [28]. On this basis, we asked whether our chimeric constructs retain G4 folding. G4Hunter analysis predicted that CA5 and MA5 contain G-rich tracts with strong potential to form stacked guanine tetrads, whereas CSA and MSA are unlikely to adopt G4 conformations (Fig. 4E) [42]. Circular dichroism spectroscopy supported these predictions. CA5 and MA5 exhibited the canonical signature of a parallel G4, with a positive band near 265 nm and a negative band around 240 nm, while CSA and MSA lacked these characteristic features (Fig. 4A–D). To assess target engagement at the cellular level, we incubated ADAMTS5-overexpressing ATDC5 cells with the tRNA-aptamer chimeras prior to immunostaining. Pre-treatment with CA5 or MA5, but not CSA or MSA, diminished ADAMTS5-specific immunofluorescence, consistent with competition between the aptamer and the antibody for the same or overlapping epitopes (Fig. 4F). ITC revealed specific sub-micromolar binding of CA5 and MA5 to recombinant ADAMTS5, with Kd values of (3.58 ± 0.51) × 10^−7^ M and (7.73 ± 0.52) × 10^−7^ M, respectively, whereas CSA and MSA showed no detectable interaction (Fig. 4G–J).

**Fig. 4:**
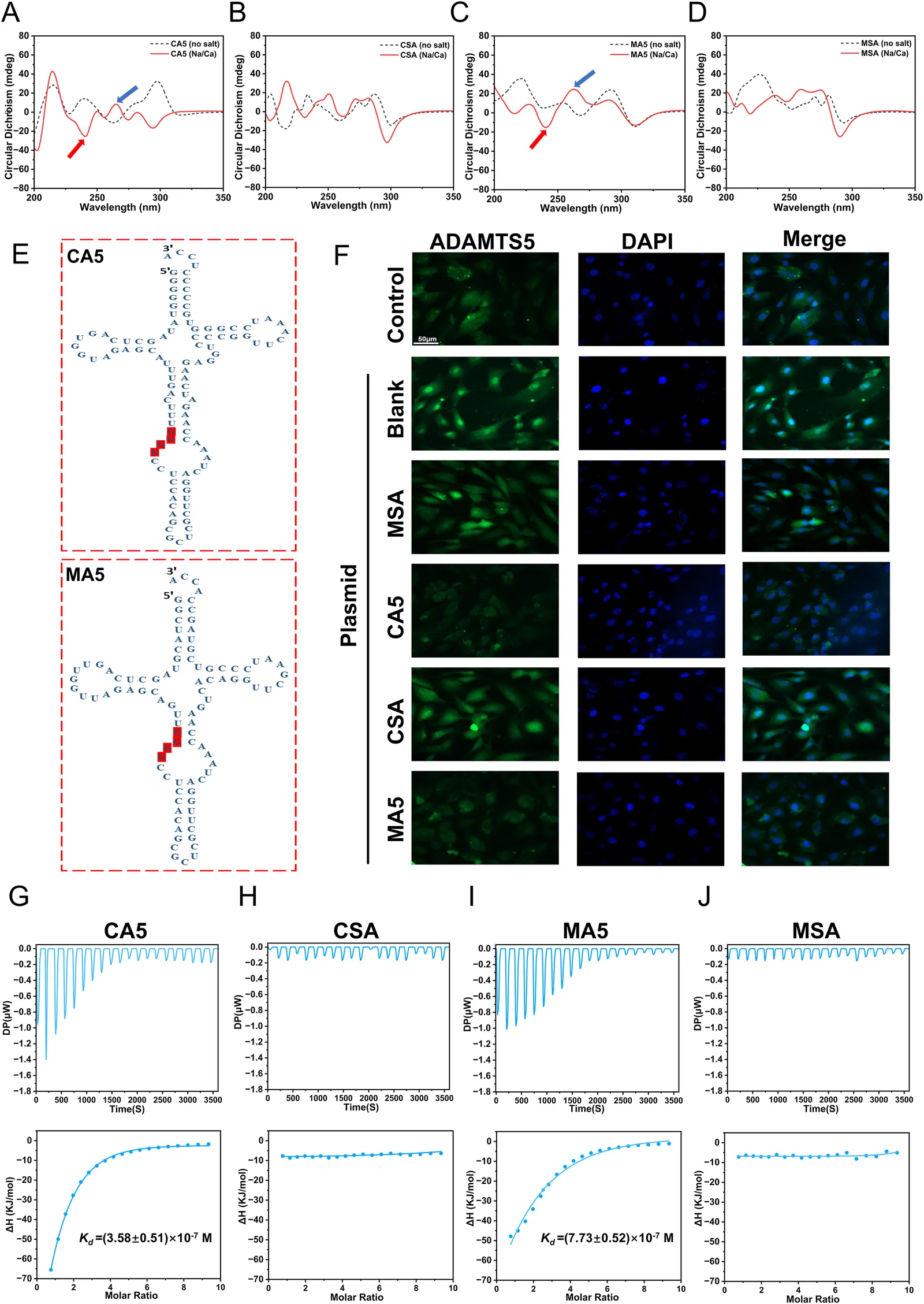
**Structural characterization and ADAMTS5 binding of tRNA-aptamer chimeras.** A-D) Circular dichroism (CD) spectra of the indicated tRNA-aptamer chimeras measured in buffers with or without metal ions (Na^+^ and Ca^2+^) to evaluate G-quadruplex (G4) folding. Red and blue arrows indicate the characteristic G4 peaks observed in CA5 and MA5, including a positive band near 265 nm (blue) and a negative band around 240 nm (red). E) G4Hunter prediction of G-quadruplex propensity for CA5 and MA5, with guanine tracts within the ADAMTS5-aptamer region highlighted in red. F) Immunofluorescence-based competition assay showing reduced ADAMTS5 antibody staining after pre-incubation with tRNA-aptamer chimeras, consistent with aptamer protein binding. G-J) Representative ITC thermograms for titration of recombinant ADAMTS5 with tRNA-aptamer chimeras. Upper, Heat flow over time; lower, Integrated heat per injection versus the molar ratio of tRNA-aptamer chimeras to ADAMTS5 protein.

Collectively, these data indicate that CA5 and MA5 adopt a G-quadruplex architecture that enables sub-micromolar binding to ADAMTS5 and robust target engagement, whereas the non-G-rich controls neither form G4 structures nor show detectable interaction with ADAMTS5, consistent with their lack of functional inhibition.

### 3.5 Transcriptome and small RNA profiling reveal a CA5/CSA-derived 3ꞌtRF that targets *Ptgs2* to suppress NF-κB signaling

To elucidate the molecular basis underlying the activity of the tRNA-aptamer chimeras, we performed mRNA sequencing in IL-1β-stimulated primary chondrocytes following transfection with CA5 or CSA. Differential expression analysis identified *Ptgs2* as one of the most consistently downregulated inflammatory transcripts in both comparisons (IL-1β vs CA5 and IL-1β vs CSA), as visualized in the volcano plots (Fig. 5A and B). Gene Ontology (GO) enrichment further indicated that DEGs (differentially expressed genes) in both datasets were strongly associated with extracellular matrix related biological processes (Fig. 5C and D). KEGG pathway analysis revealed significant enrichment of NF-κB signaling in the CA5 and CSA transcriptomes (Fig. 5E and F), suggesting that convergent regulation of the *Ptgs2*/NF-κB axis may represent a shared anti-inflammatory program engaged by these two constructs. We next investigated whether intracellular processing of the tRNA-aptamer chimeras generates functional tRNA-derived fragments that could account for this transcriptional signature. Small RNA sequencing showed that CA5 and CSA were predominantly processed into canonical tRF species, including 5′tRFs, 3′tRFs, and internal tRFs (i-tRFs) (Fig. 5G and H). Using miRNA target prediction (miRDB), we identified a shared 3′end tRNA-derived fragment generated from CA5/CSA (3′tRF; 5′-GTTCAAATCCGGGTGCCCCCTCCA-3ꞌ) with predicted complementarity to the 3′UTR of *Ptgs2*. Consistent with this prediction, small RNA-seq showed that the abundance of 3ꞌtRF was reduced under IL-1β challenge but was markedly increased after CA5 or CSA transfection (Fig. 5I). Integrative intersection analysis combining predicted 3ꞌtRF targets, CA5/CSA-regulated DEGs, and a curated pyroptosis associated gene set [43], [44], [45] identified *Ptgs2* as the overlapping candidate (Fig. 5J). We then validated this regulatory relationship experimentally. RT-qPCR and WB confirmed that IL-1β robustly induced *Ptgs2* mRNA and protein, whereas CA5 or CSA significantly suppressed both readouts; in contrast, MA5 did not measurably affect *Ptgs2* expression (Fig. 5K and L). In parallel, WB analysis of NF-κB pathway activation showed reduced phosphorylation of p65 and IκBα in CA5 and CSA treated cells (Fig. 5M–O). Consistently, immunofluorescence microscopy revealed prominent IL-1β driven nuclear accumulation of p65 in IL-1β and MSA treated chondrocytes, which was reversed by CA5 or CSA, while MA5 failed to prevent p65 nuclear translocation (Fig. 5P and Q).

**Fig. 5:**
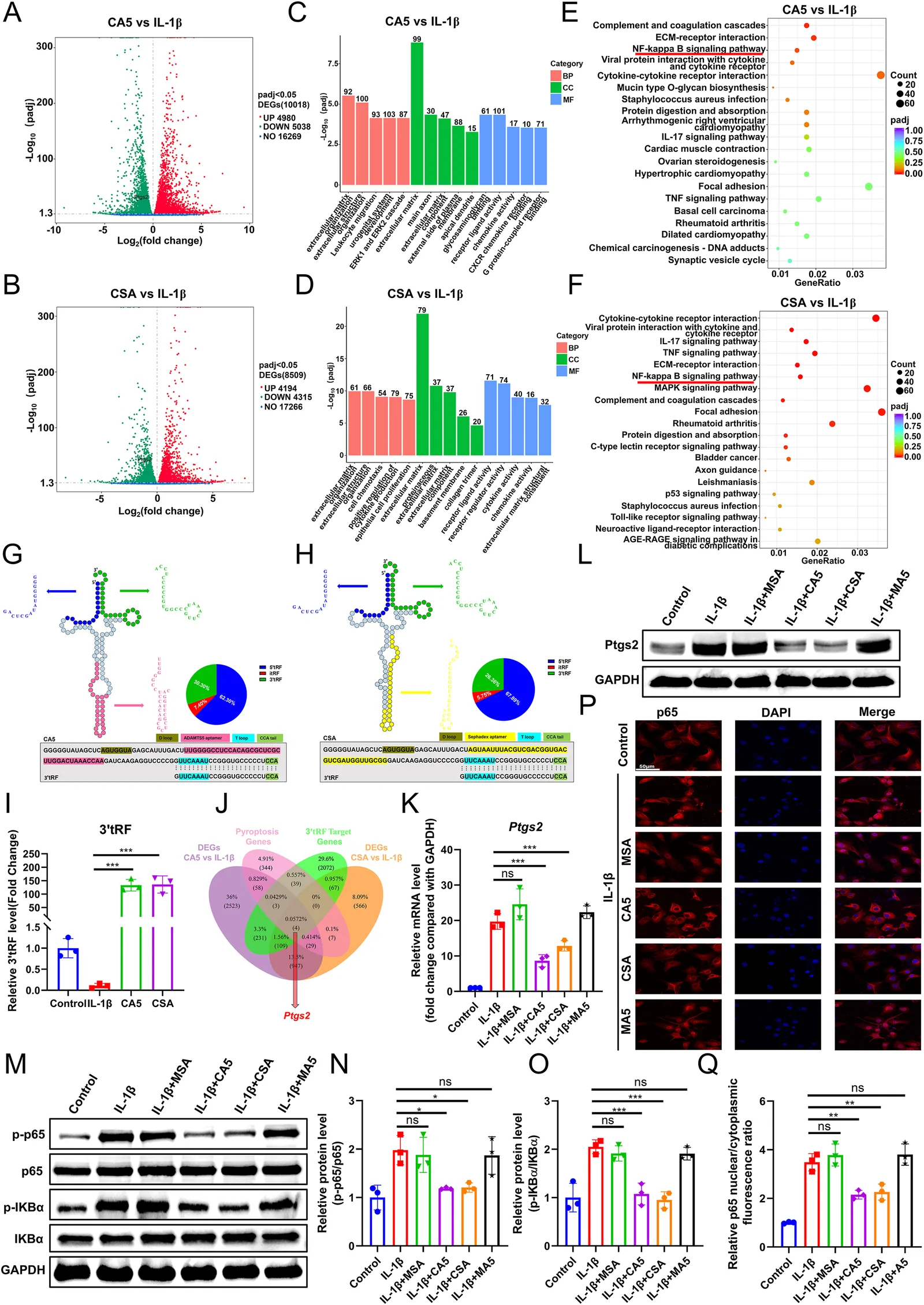
**Integrated mRNA and small RNA profiling identifies a chimera-derived 3ꞌtRF that targets *Ptgs2* and dampens NF-κB signaling.** A-B) Volcano plots of DEGs in IL-1β-stimulated primary chondrocytes following transfection with CA5 or CSA, highlighting *Ptgs2*. C-D) GO enrichment analysis of DEGs from IL-1β vs CA5 and IL-1β vs CSA comparisons. E-F) KEGG pathway enrichment analysis of DEGs from IL-1β vs CA5 and IL-1β vs CSA comparisons, showing enrichment of NF-κB signaling. G-H) Small RNA-seq mapping profiles of reads aligned to CA5 and CSA, revealing predominant processing into 5ꞌtRFs, 3ꞌtRFs, and i-tRFs. I) Abundance of the 3ꞌtRF (sequence: 5ꞌ-GTTCAAATCCGGGTGCCCCCTCCA-3ꞌ) in IL-1β-stimulated primary chondrocytes with or without CA5/CSA transfection, quantified from small RNA-seq. J) Venn diagram showing overlap among predicted targets of the 3ꞌtRF, CA5/CSA-regulated DEGs, and a curated pyroptosis related gene set, identifying *Ptgs2* as the intersecting candidate. K-L) RT-qPCR and Western blot validation of *Ptgs2* expression in IL-1β-stimulated primary chondrocytes after transfection with CA5, CSA, or MA5. M − O) Western blot analysis of NF-κB pathway activation, including phosphorylated and total p65 and IκBα, following the indicated treatments. P) Immunofluorescence images of p65 nuclear localization in IL-1β-stimulated primary chondrocytes transfected with the indicated constructs. Q) Quantification of p65 nuclear translocation based on the nuclear-to-cytoplasmic fluorescence intensity ratio measured using ImageJ. Data were presented as mean ± SD (n = 3). Statistical analysis was performed using one-way ANOVA followed by Tukey's test. *P < 0.05 **P < 0.01 and ***P < 0.001 vs. the IL-1β group.

Together, these findings support a model in which CA5 and CSA are intracellularly processed to generate a conserved 3ꞌtRF that targets *Ptgs2*, thereby repressing *Ptgs2* expression and dampening NF-κB activation. This mechanism links chimera-derived fragment processing to coordinated regulation of inflammatory signaling and pyroptosis related programs in chondrocytes.

### 3.6 3ꞌtRF directly targets the *Ptgs2* 3ꞌ-UTR to suppress NF-κB activation and preserve ECM

To define how the 3′tRF regulates *Ptgs2*, we inspected the 3′UTR of mouse *Ptgs2* and identified a putative interaction motif (UUUGAAA), which was mutated to AAACUUU for validation (Fig. 6A). Dual-luciferase assays showed that CA5, CSA, or a synthetic 3ꞌtRF mimic significantly reduced reporter activity driven by the wild-type *Ptgs2* 3ꞌUTR, whereas the mutant reporter abolished these effects (Fig. 6B and C), indicating a direct, site-dependent interaction. In IL-1β-stimulated primary chondrocytes, transfection of the 3ꞌtRF mimic, either alone or combined with the ADAMTS5 aptamer (A5), significantly attenuated IL-1β-induced *Ptgs2* mRNA and protein upregulation, while A5 alone had minimal impact (Fig. 6D and E). Consistently, immunoblotting revealed reduced phosphorylation of p65 and IκBα in the 3ꞌtRF and 3ꞌtRF + A5 groups, whereas A5 alone showed little change (Fig. 6F–H). Immunofluorescence further confirmed that the 3ꞌtRF, with or without A5, reversed IL-1β-driven p65 nuclear accumulation, whereas A5 failed to block p65 translocation (Fig. 6I and J). Functionally, Toluidine blue and Safranin-O staining demonstrated the strongest restoration of ECM retention with 3ꞌtRF + A5, followed by the 3ꞌtRF alone and A5 alone, while the scrambled control was ineffective (Fig. 6K–M).

**Fig. 6:**
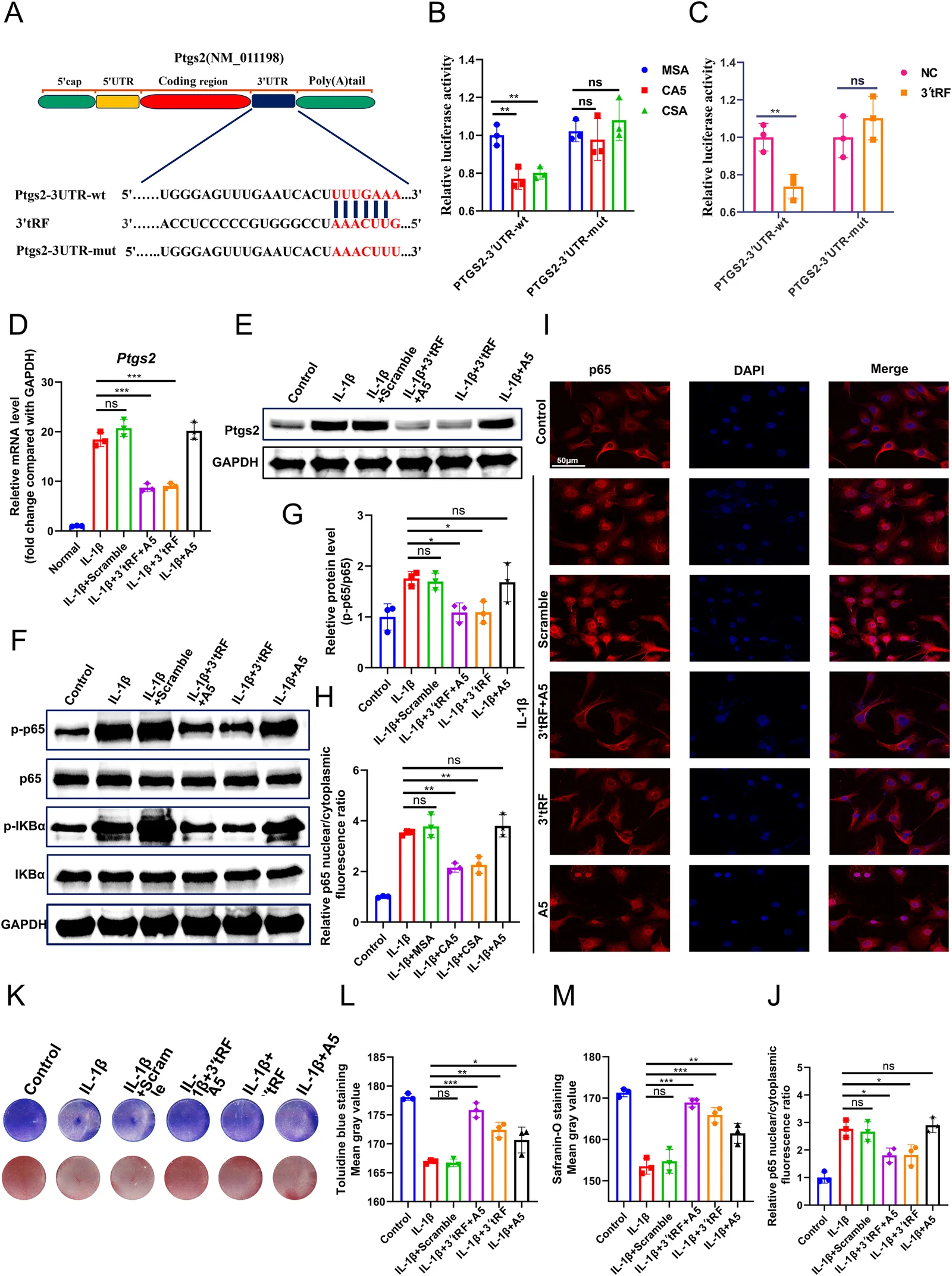
**3ꞌtRF derived from CA5 and CSA targets *Ptgs2* 3ꞌUTR to suppress NF-κB activation and protect ECM in IL-1β-stimulated primary chondrocytes.** A) Schematic illustrating prediction of the 3ꞌtRF binding site in the *Ptgs2* 3ꞌUTR and the corresponding mutant design. B-C) Dual-luciferase reporter assays showing the luciferase activity of wild-type or mutant *Ptgs2* 3ꞌUTR constructs upon transfection with CA5, CSA, or 3ꞌtRF mimic. D-E) RT-qPCR and Western blot analysis of *Ptgs2* mRNA and protein expression in IL-1β-stimulated primary chondrocytes transfected with 3ꞌtRF mimic, ADAMTS5 aptamer (A5), or their combination. F-H) Western blot analysis of NF-κB pathway activation showing reduced phosphorylation of p65 and IκBα in primary chondrocytes treated with 3ꞌtRF + A5 or 3ꞌtRF alone. I) Immunofluorescence images showing p65 nuclear translocation in IL-1β-stimulated chondrocytes treated with the indicated constructs. J) Quantification of p65 nuclear translocation based on the nuclear-to-cytoplasmic fluorescence intensity ratio measured using ImageJ. K-M) Toluidine blue and Safranin-O staining of ECM in IL-1β-stimulated primary chondrocytes transfected with 3ꞌtRF + A5, 3ꞌtRF, A5, or scrambled control, showing ECM retention. Data were presented as mean ± SD (n = 3). Statistical analysis was performed using one-way ANOVA followed by Tukey's test. *P < 0.05 **P < 0.01 and ***P < 0.001 vs. the IL-1β group or the corresponding negative control (NC or MSA, as indicated).

Collectively, these data establish a direct 3ꞌtRF/*Ptgs2* 3ꞌUTR interaction and support a mechanism in which the chimera-derived 3ꞌtRF suppresses *Ptgs2* expression and NF-κB signaling, thereby contributing to protection of cartilage matrix homeostasis.

## 4 Discussion

Osteoarthritis is increasingly recognized as a whole-joint disease driven by interconnected catabolic and inflammatory programs, which may help explain why many single-target strategies have failed to achieve durable disease modification in clinical studies [46], [47]. RNA-based therapeutics, including aptamers, siRNAs, and tRNA-derived fragments, offer a modular framework for integrating extracellular target blockade with intracellular gene regulation within a single molecular construct [48], [49], [50]. Building on this rationale, we designed CA5 to couple preservation of cartilage matrix integrity with an additional intracellular regulatory layer, thereby simultaneously constraining matrix breakdown and the inflammatory cell-death amplification that perpetuates tissue injury. Consistent with this concept, CA5 exhibited marked module synergy and conferred robust chondroprotection in IL-1β-stimulated chondrocytes, preserving cartilage matrix, attenuating inflammatory responses, and strongly suppressing pyroptosis. Moreover, intra-articular administration of CA5 mitigated cartilage degeneration and overall joint pathology in the DMM model. Collectively, these findings support the notion that coordinated multi-target intervention may be required to achieve durable disease modification in OA.

Mechanistic interrogation revealed that the tRNA^Cys^ scaffold is processed into a conserved 24 nt 3ꞌtRF that represses *Ptgs2* by directly targeting its 3ꞌUTR in a miRNA-like manner, consistent with emerging OA-relevant tRF mechanisms such as mt-tRF3b targeting *Senp1*, tRF365 suppressing *Ikbkb*, tRF3031B silencing *Rela* expression and tRF16 binding to *ALKBH5* [35], [51], [52], [53]. Re-analysis of our small RNA-seq data showed that the 24 nt 3ꞌtRF identified here represents the predominant member of a nested tRNA^Cys^-derived 3ꞌtRF family, whereas tRF-3003a is a shorter and less abundant nested fragment (Fig. S5). Although these fragments share a substantial downstream core sequence, tRF-3003a does not retain the complete upstream GUUCAAA region corresponding to the *Ptgs2*-interacting site, supporting our focus on the predominant 24 nt species. Green et al. reported that tRF-3003a was transiently induced during the early phase of IL-1β stimulation and subsequently declined, whereas the 24 nt tRNA^Cys^-derived 3ꞌtRF identified here was reduced after 24 h of IL-1β exposure [33]. These observations are not necessarily contradictory, because the reduced abundance of tRF-3003a in OA cartilage reflects a chronic disease state, whereas its early induction after IL-1β exposure represents a transient response to acute inflammatory stimulation. This difference may reflect both the temporal dynamics of the inflammatory stress response and fragment-specific processing of tRNA^Cys^-derived 3ꞌtRFs. PTGS2 is a central inducible mediator within OA cytokine networks, functioning both as a canonical NF-κB responsive effector gene and as a key driver of prostaglandin-dependent inflammatory amplification [2], [54], [55]. In the chronically inflamed OA joint microenvironment, sustained cytokine exposure maintains an NF-κB dominant transcriptional program that correlates with pain and structural progression [56]. In this setting, NF-κB driven upregulation of *Ptgs2* increases COX-2 derived Prostaglandin E2 (PGE2) production, which in turn signals through E prostanoid rhodopsin-like G-protein coupled receptors (EP1-4), particularly EP4, to promote p65 phosphorylation and reinforce NF-κB activity, establishing a feed-forward loop that perpetuates inflammatory and catabolic gene expression [57], [58]. Therefore, by suppressing *Ptgs2*, the chimera-derived 3′tRF is expected to reduce PGE2-EP4 signaling and weaken the persistence of NF-κB activation, thereby limiting inflammasome priming and inflammasome-associated transcription in chondrocytes and ultimately improving OA outcomes [59], [60], [61], [62]. Consistent with this mechanism, CSA retained significant matrix-protective activity compared with MSA despite lacking functional ADAMTS5-binding activity, likely reflecting the independent contribution of the tRNA^Cys^-derived 3ꞌtRF in suppressing inflammation-associated ECM degradation. The greater potency of CA5 therefore likely results from the combined effects of scaffold-derived intracellular regulation and direct ADAMTS5 inhibition.

A defining feature of tRNA-aptamer chimera is that the tRNA scaffold functions as more than a structural linker; it serves as an active module that can be physiologically processed into regulatory tsRNAs with intracellular effector activity [63]. Owing to its conserved secondary/tertiary architecture and abundant base modifications, tRNA provides a compact, endogenous-like framework that can enhance folding, nuclease resistance, and intracellular handling relative to naked small RNAs, thereby expanding the design space for RNA therapeutics [64]. In this paradigm, the effector sequence is encoded within the scaffold and released through endogenous processing (e.g., generation of 3ꞌtRFs from the T-loop by Dicer and/or angiogenin), enabling Argonaute-associated gene silencing and coordinated suppression of pathological pathways [33], [65]. This built-in processing logic provides a mechanistically coherent explanation for how a single chimeric RNA can couple extracellular engagement to intracellular pathway rewiring. A second design advantage is that the tRNA framework can serve as a structural carrier that supports presentation of the aptamer domain without erasing its functional fold. In CA5 and MA5, the aptamer module retains a G-quadruplex (G4)-containing architecture and measurable ADAMTS5 engagement, whereas scaffold-only controls lacking the G4 motif show no detectable binding, consistent with prior reports that G4 structures can confer high-affinity ADAMTS5 binding and functional inhibition [27], [28]. Importantly, this implies that the chimera architecture can be engineered such that tRF biogenesis occurs from the scaffold while the aptamer maintains its native higher-order conformation, allowing the two modules to operate in parallel rather than compromising one another. Beyond mechanism and structure, tRNA-aptamer chimeras offer a practical manufacturing advantage because they can be produced at high levels in *E. coli*, leveraging the intrinsic stability and transcriptional robustness of tRNA-based transcripts [37], [66]. This fermentation-style production can, in principle, enable cost-effective scale-up, improved batch-to-batch consistency, and rapid design build iteration compared with fully synthetic oligonucleotide workflows, provided that purification and quality controls such as endotoxin removal, integrity and processing profiling, and impurity testing are rigorously implemented [67]. Collectively, programmable tRF release, preserved aptamer G4 folding with sustained target engagement, and scalable bacterial expression support tRNA-aptamer chimeras as a versatile and programmable therapeutic modality that couples durable extracellular neutralization with tunable intracellular gene regulation to address the multi-factorial circuitry of OA.

In parallel to the intracellular *Ptgs2*-silencing arm, CA5 incorporates an ADAMTS5-directed aptamer, a short single-stranded nucleic acid that folds into a defined three-dimensional structure and binds protein targets with antibody-like specificity, most often acting as a competitive antagonist [68]. ADAMTS5 is widely recognized as a principal aggrecanase that drives early aggrecan loss and proteoglycan depletion in OA, and genetic inactivation of ADAMTS5 markedly protects cartilage in instability-induced OA models, supporting this protease as an upstream, disease-relevant node for matrix preservation [69]. Nonetheless, clinical experience highlights the limitations of single-target aggrecanase inhibition, as a phase 2 randomized trial reported that the oral ADAMTS5 inhibitor S201086/GLPG1972 did not significantly reduce MRI-assessed cartilage loss or improve symptoms over 52 weeks, consistent with the concept that heterogeneous OA endotypes can remain sustained by parallel inflammatory and catabolic programs despite extracellular protease blockade [3], [70], [71]. Beyond structural preservation, accumulating evidence links ADAMTS5-driven matrix breakdown to innate immune activation, because aggrecan and related cartilage metabolites can function as DAMP-like signals that engage TLR2-dependent pathways; notably, an aggrecan 32-mer fragment can drive OA pain via TLR2, and ADAMTS5-generated cartilage fragments can activate TLR2 in vitro, an effect that is attenuated by anti-ADAMTS5 biologic intervention [25], [72], [73], [74]. This may also help explain why MA5 attenuated pyroptotic responses despite showing no measurable suppression of NF-κB activation. Although ECM-derived fragments can initiate innate immune signaling, a direct causal link between ADAMTS5-generated matrix fragments and NLRP3 inflammasome activation has not been conclusively established. These matrix-derived signals may contribute to inflammasome priming, whereas additional cellular stress signals, such as oxidative stress and ionic disturbances, may participate in subsequent inflammasome activation [75], [76]. Thus, the anti-pyroptotic effect of MA5 is more appropriately interpreted as an indirect consequence of ECM preservation rather than direct inflammasome inhibition. In this context, the aptamer module is expected not only to limit aggrecan cleavage but also to reduce the production of pro-inflammatory matrix cues that amplify joint inflammation and pain, thereby providing mechanistic complementarity to CA5's intracellular anti-inflammatory program.

Collectively, CA5 demonstrates that a single tRNA-aptamer chimera can integrate selective extracellular protease blockade with endogenous tRF-driven gene silencing to achieve multi-node suppression of OA pathology. A working model is summarized in Fig. 7. This platform offers a generalizable route to disease modification by jointly targeting matrix breakdown and inflammatory amplification while motivating future studies on durability, safety, and translational efficacy across OA endotypes.

**Fig. 7:**
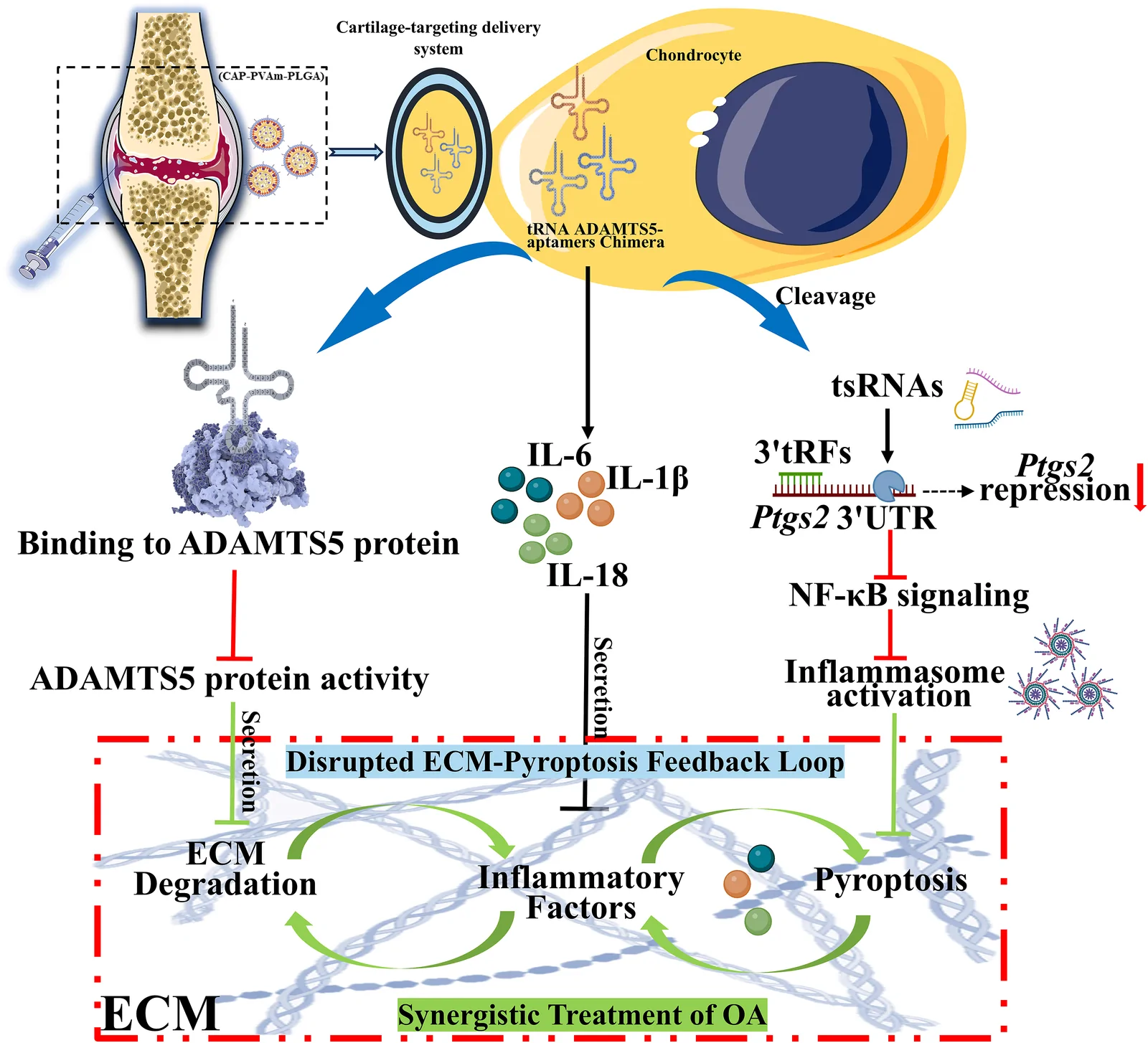
**Working model.** Cartilage-targeted CAP-PVAm-PLGA delivers tRNA^Cys^ aptamer chimera to OA chondrocytes, where processing generates a 3′tRF that interacts with the *Ptgs2* 3ꞌUTR and suppresses *Ptgs2* expression, thereby attenuating NF-κB signaling and inflammasome activation. In parallel, the aptamer domain inhibits ADAMTS5-mediated ECM degradation, together disrupting the ECM-pyroptosis feedback loop and ameliorating OA.

## CRediT authorship contribution statement

**Bo Sang:** Writing – review & editing, Writing – original draft, Validation, Data curation, Conceptualization. **Ye Tian:** Writing – review & editing, Writing – original draft, Funding acquisition. **Kang Ru:** Data curation. **Xuehao Wang:** Methodology. **Suryaji Patil:** Data curation. **Wenjin Zhong:** Data curation. **Xinyao Jia:** Data curation. **Zhihao Chen:** Data curation. **Hui Li:** Writing – review & editing. **Airong Qian:** Supervision, Project administration.

## Availability of data and materials

The datasets generated and analyzed during the current study are available from the corresponding author upon reasonable request.

## Ethics approval and consent to participate

The experimental procedures for animal care and use were conducted in accordance with the Guide for the Care and Use of Laboratory Animals and were approved by the Laboratory Animal Ethics and Welfare Committee of Northwestern Polytechnical University (Approval No. 202301106).

## Funding

The authors disclose receipt of the following financial or material support for the research, authorship, and/or publication of this article: This study was supported by the 10.13039/501100001809National Natural Science Foundation of China (32371296) and the Shaanxi Provincial Funding Program for Scientific Activities of Returned Scholars (24004). The funders had no role in the study design, data collection, analysis or interpretation, writing of the manuscript, or the decision to submit the article for publication.

## Declaration of competing interest

The authors declare that they have no known competing financial interests or personal relationships that could have appeared to influence the work reported in this paper.

## Figures and Tables

**figs1:**
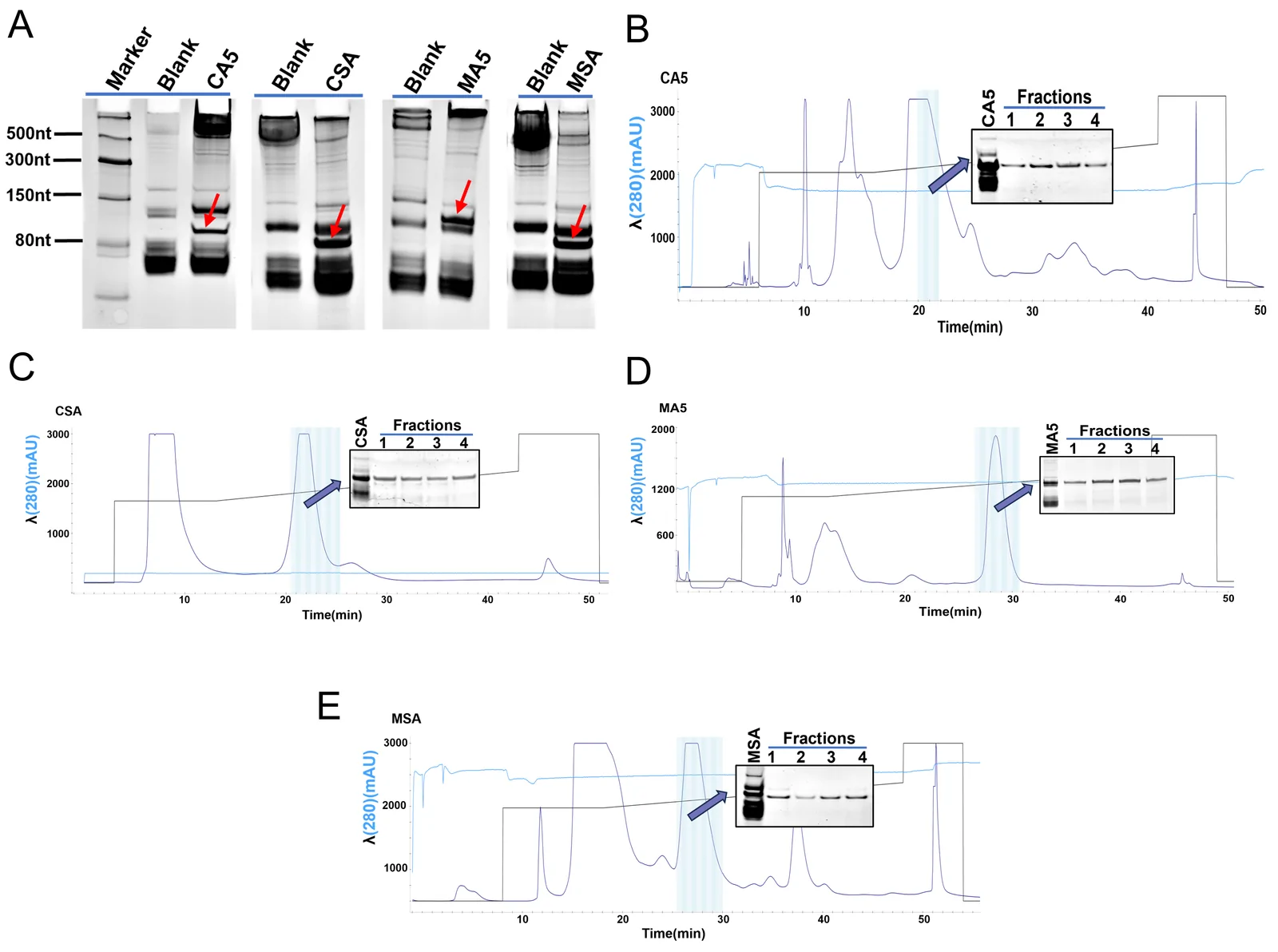


**figs2:**
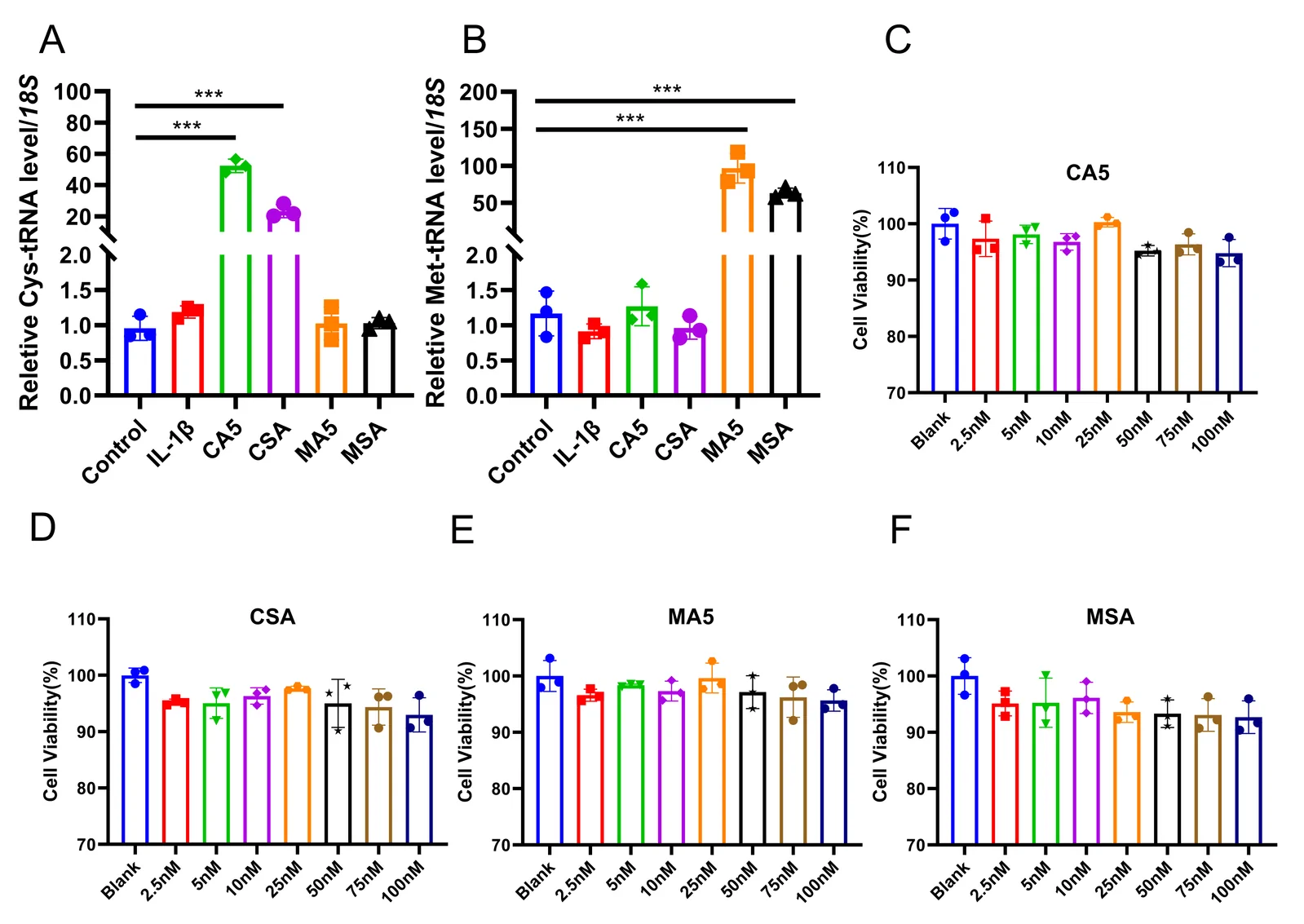


**figs3:**
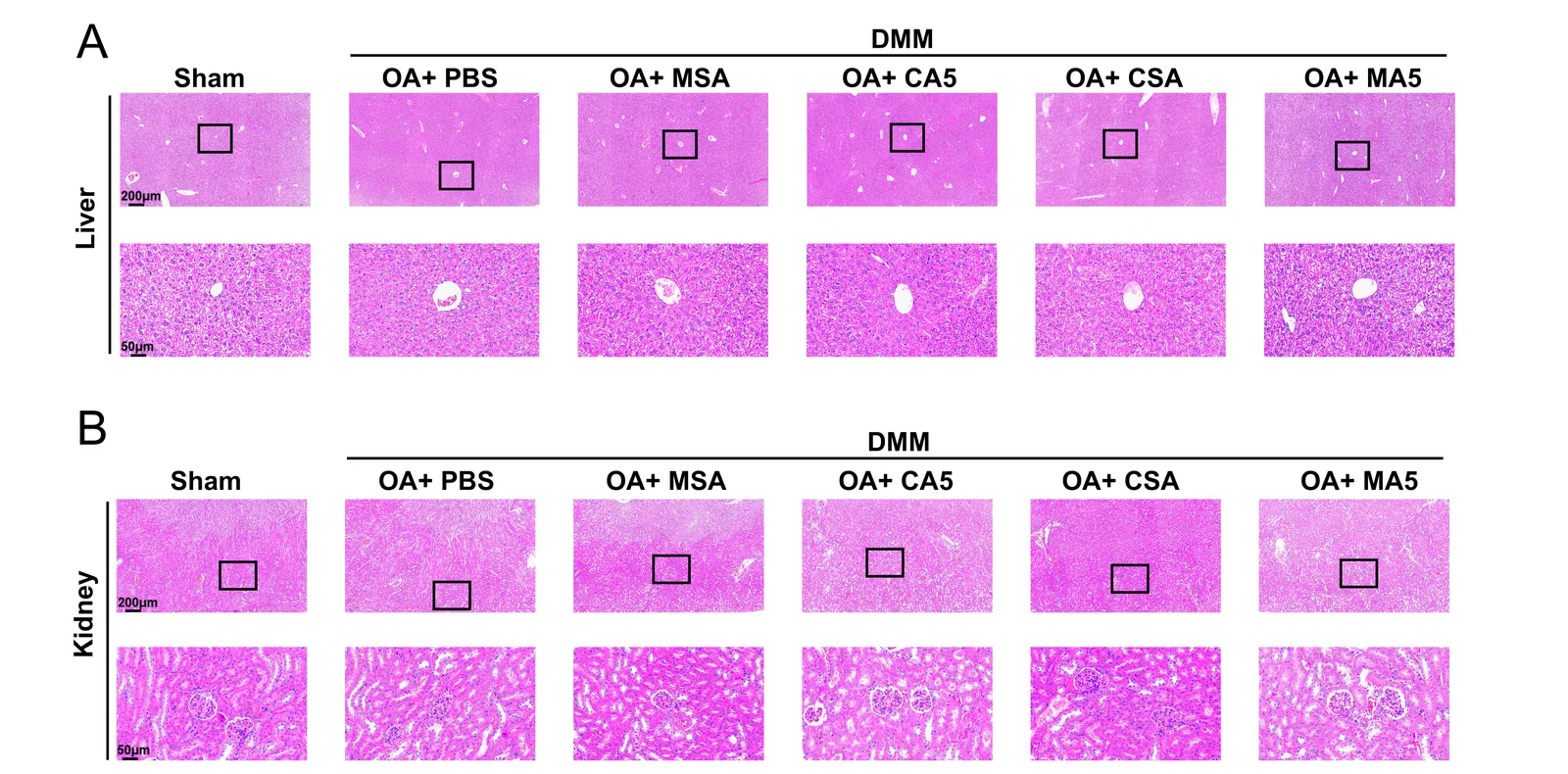


**figs4:**
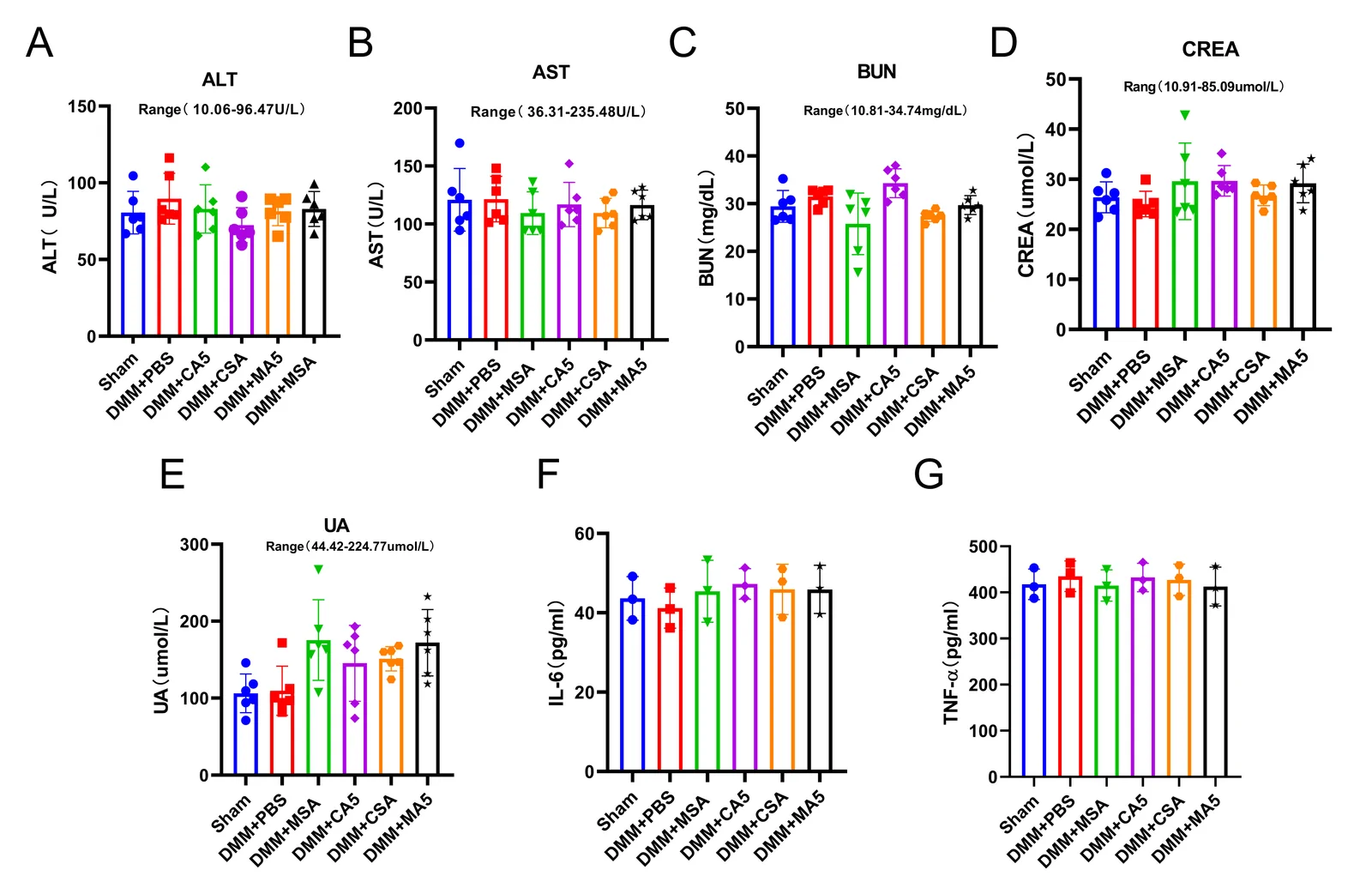


**figs5:**