# Supplementary material for: A tRNACys-aptamer chimera co-targeting ADAMTS5 and Ptgs2 interrupts the ECM-pyroptosis feedback loop in osteoarthritis
Source: Noncoding RNA Res. 2026 Nov 3;21:57–73. doi: 10.1016/j.ncrna.2026.10.003 (PMC13756542; doi:10.1016/j.ncrna.2026.10.003)
Supplement: Supplementary file 1 [file mmc1.docx]

**Figure S1**. Bacterial expression, and purification of tRNA-aptamer chimeras. A) tRNA-aptamer chimeras were produced in *E. coli* and analyzed by denaturing urea-PAGE. Total RNA isolated from untransformed HST08 cells served as the negative control. Red arrows mark prominent bands corresponding to highly expressed tRNA-aptamer chimeras. B-E) Representative FPLC profiles (UV detection) of tRNA-aptamer chimeras extracted from *E. coli* showing purification/enrichment of the target products. Insets: urea-PAGE analysis of collected fractions (#1~#4), verifying the fractions containing the desired tRNA-aptamer chimeras.

**Figure S2**. Intracellular expression and cytocompatibility of tRNA-aptamer chimeras. A-B) Following transfection, strong intracellular accumulation of the indicated tRNA-aptamer chimeras was detected, confirming efficient cellular delivery and expression. C-F) Cell viability assessed by CCK-8 demonstrated that tRNA-aptamer chimeras exhibited no detectable cytotoxicity across the tested concentration range, remaining well tolerated up to 100nM. Data were shown as the mean ± SD and statistical significance was calculated by one-way ANOVA followed by Tukey's test. **P*<0.05, ***P*<0.01 and ****P* < 0.001 vs. the control group.

**Figure S3.** Systemic safety assessment of tRNA-aptamer chimeras. A-B) Representative H&E-stained sections of liver and kidney collected from animals receiving the indicated treatments.

**Figure S4** Serum biochemistry and inflammatory markers after treatment. A-E) Serum biochemical indices (ALT, AST, BUN, CREA, UA) were measured to assess systemic safety; no treatment-related alterations were detected (mean ± SD, n = 6). F-G) Serum pro-inflammatory cytokines (IL-6, TNF-α) were quantified, showing no abnormal changes across groups (mean ± SD, n = 3).

**Figure S5 Sequence, positional distribution, and abundance of major tRNA^Cys^-derived 3ꞌtRFs from CA5 and CSA.** A-B) Sequence alignment and small RNA-seq read counts of major tRNA^Cys^-derived 3ꞌtRF species generated from CA5 (A) and CSA (B). The predominant 24nt 3ꞌtRF (5ꞌ-GUUCAAAUCCGGGUGCCCCCUCCA-3ꞌ; positions 85-108), its closely related isoforms, the previously reported tRF-3003a (5ꞌ-UCCGGGUGCCCCCUCCA-3ꞌ; positions 92-108), and related shorter isoforms are shown according to their positions within the parental tRNA^Cys^-containing constructs. The GUUCAAA region corresponding to the experimentally validated *Ptgs2*-interacting site is indicated. Read counts are presented as mean ± SD (n = 3).

**Supplementary Table 1. Primer sequences**

| Gene | Sequence (5'-3') |
| --- | --- |
| GAPDH | Forward: CCTCTGACTTCAACAGCGAC  Reverse: TCCTCTTGTGCTCTTGCT GG |
| U6 | Forward: GTGCTCGCTTCGGCAGCACATAT  Reverse: AAAATATGGAACGCTTCACGAA |
| tRNA^Cys^ | Forward: GGGGGTATAGCTCAGTGGTA  Reverse: TGGAGGGGGCACCCGGATTT |
| tRNA^Met^ | Forward: GGCTACGTAGCTCAGTTGGT  Reverse: TGGTGGCTACGACGGGATTC |
| Col2a1 | Forward: ACACCGCTAACGTCCAGATG  Reverse: TCGGTACTCGATGACGGTCT |
| Aggrecan | Forward: GGAGACCCAGACAGCAGAAACAAC  Reverse: GCAGGTGGCTCCATTCAGACAAG |
| Ptgs-2 | Forward: CTGGTGCCTGGTCTGATGATGTATG  Reverse: GGATGCTCCTGCTTGAGTATGTCG |
| Il-6 | Forward: GGAGCCCACCAAGAACGATAG  Reverse: GTGAAGTAGGGAAGGCCGTG |
| Nlrp3 | Forward: CCAGACCTCCAAGACCACTACG  Reverse: CAGAGAAGAGATGCTCCTCAATGC |
| Caspase-1 | Forward: AATATGCCTGGTCTTGTGACTTGG  Reverse: CATCTGTAGCCTAAATTCTGGTTGTTC |
| Asc | Forward: CCACCAATGCCTTCGCCTTCTG  Reverse: GGTAGGTTCTTGCGTGGGTCAAC |
